# Epidemiology of Craniomaxillofacial Trauma in Chile: A Systematic Review and 24-Year Nationwide Interrupted Time-Series Analysis

**DOI:** 10.3390/cmtr19030032

**Published:** 2026-07-03

**Authors:** Gustavo Sáenz-Ravello, Paula Carrasco García, Laura Sáenz-Ravello, Elda L. Fisher

**Affiliations:** 1 Center for Surveillance and Epidemiology of Oral Diseases (CEVEO), Faculty of Dentistry, University of Chile, Olivos 943, Independencia, Santiago 8380544, Chile; 2 Servicio de Salud Tarapacá, Ministerio de Salud, Anibal Pinto 815, Iquique 1100527, Chile; 3 School of Health and Wellbeing, Health Economics and Health Technology Assessment, College of Medical Veterinary and Life Sciences, University of Glasgow, Glasgow G12 8TB, UK; 4 School of Nursing, Facultad de Medicina, Pontificia Universidad Catolica de Chile, Santiago 8331150, Chile; 5 Division of Plastic, Maxillofacial, and Oral Surgery, Department of Surgery, Duke University Medical Center, Durham, NC 27710, USA; elda.fisher@duke.edu

**Keywords:** Craniomaxillofacial trauma, epidemiology, Chile, systematic review, meta-analysis, interrupted time series analysis

## Abstract

Craniomaxillofacial trauma (CMFt) poses a significant burden, yet in many countries the evidence base is fragmented across single-center hospital series without specialized registry. Using Chile as a case study, we demonstrate a dual-synthesis approach to construct a national CMFt profile. Six databases were searched through February 2026 (PROSPERO: CRD420261290860). Two reviewers independently screened studies. Risk of bias was assessed with the JBI critical appraisal tool. Fracture-site proportions were pooled via random-effects meta-analysis and synthesized using GRADE. DEIS trauma discharges (2001–2024) were analyzed with negative binomial interrupted time-series. Nineteen studies were included. CMFt represented 2.6–6.1% of emergency consultations. CMFt admissions were 54.2/1000 trauma discharges; this rate dropped during 2020–2021 and rebounded post-2022. Pooled fracture-site distributions were highest for mandibular (45.3%) and zygomatic (24.2%) fractures. CMFt disproportionately affected males across both hospital series and national discharge data. According to DEIS, low-energy accidental injuries were the predominant etiology, followed by transport-related high-energy injuries and interpersonal violence, contrasting with hospital series where interpersonal violence predominated among adult surgical cohorts. Fracture admissions had longer length of stay (LOS) than soft-tissue CMFt (+0.94 days), with mean LOS ranging from 2.08 (nasal) to 8.35 days (multiple skull/facial fractures). These findings support prioritizing surgical preparedness and training in common fracture patterns, while strengthening trauma surveillance, referral pathways, and service planning in health systems without dedicated CMFt registries.

## 1. Introduction

Craniomaxillofacial trauma (CMFt) comprises a heterogeneous group of injuries affecting the facial skeleton and associated soft tissues [1], with consequences ranging from acute functional impairment (occlusion, mastication, airway compromise, and vision) to long-term esthetic sequelae and psychosocial burden [2]. Beyond clinical morbidity, CMFt represents a relevant driver of emergency care utilization, inpatient admissions, operative workload, and length of stay (LOS), making it a meaningful indicator of trauma system demand for oral and maxillofacial surgery services [3,4,5]. As a result, service planning strategies require understanding the distribution of fracture sites and mechanisms to anticipate caseload complexity, allocate operating room resources, and inform prevention priorities [6].

The epidemiology of CMFt is strongly shaped by the distribution of external causes, including interpersonal violence, transport-related injuries, accidental falls, and occupational mechanisms, as well as by demographic changes and contextual factors that can vary markedly across regions and over time [7]. Prior systematic reviews indicate that CMFt accounts for a meaningful share of emergency presentations and that etiologic patterns vary widely across settings, driven by contextual and sociodemographic factors [7,8,9]. This cross-country variability underscores the need for country-specific syntheses with consistent definitions and denominators, particularly in settings where the evidence is fragmented across single-center series. Indeed, in many health systems, dedicated CMFt registries are absent, and national profiles must be assembled from heterogeneous hospital series supplemented by administrative discharge data, a methodological challenge that is common across Latin America, yet for which no standardized analytical template has been established.

Chile illustrates this challenge well. The available evidence on CMFt has primarily been generated from hospital-based observational series, often from single institutions, with heterogeneous inclusion criteria and reporting practices [10,11,12,13,14,15,16,17,18,19,20,21,22,23,24,25,26,27,28]. Some studies restrict inclusion to specific fracture types or to surgically treated patients, whereas others focus on emergency presentations or inpatient cohorts, frequently without a shared denominator. Consequently, estimates are not always comparable because denominators vary, and many reports are most informative for describing patterns among CMFt cases rather than quantifying CMFt occurrence within a broader healthcare population. This fragmentation limits the ability to derive pooled estimates that are directly actionable for national-level planning and obscures whether observed differences reflect true epidemiologic variation versus differences in study design and case definition [29].

A systematic review and meta-analysis (SR/MA) can consolidate fragmented hospital literature by pooling comparable outcomes and by synthesizing fracture-site and etiologic distributions across hospital series. However, reliance on the published hospital literature alone may still be limited by geographic concentration, small samples, incomplete temporal coverage, and selection mechanisms tied to referral pathways and admission thresholds. Complementary analyses using nationwide administrative hospital discharge data can therefore strengthen external validity by capturing long-run trends and hospitalization burden, particularly when the data source provides consistent coding across years and across a broad range of institutions. This dual-synthesis approach offers a replicable analytical template for any country that possesses ICD-coded discharge databases but lacks a dedicated trauma registry [30].

Accordingly, using Chile as a case study, this SR/MA aimed to synthesize the CMFt patterns reported in hospital-based settings, including pooled distributions of major fracture sites and reported etiologic profiles. In addition, we contextualized the evidence synthesis with a complementary nationwide analysis of Chilean hospital discharge data (DEIS, 2001–2024) to describe temporal trends in CMFt admissions and hospitalization burden (LOS). By integrating these sources, we sought to provide an updated, clinically relevant epidemiologic profile to inform prevention priorities and trauma service planning, while demonstrating a methodological framework applicable to other countries with a similar data infrastructure but without dedicated CMFt registries.

## 2. Materials and Methods

### 2.1. Protocol and Study Design

This SR was conducted following the Joanna Briggs Institute (JBI) guidelines for the SR of observational epidemiological studies reporting prevalence and cumulative incidence data [31]. The protocol for this SR was prospectively registered in PROSPERO (CRD420261290860) and complied with the PRISMA checklist for SR [32]. The original protocol was not amended.

### 2.2. Eligibility Criteria

This review addressed the following question: What is the prevalence and what are the epidemiologic patterns of CMFt among patients in Chilean healthcare settings?

*Population:* We included studies of patients who attended Chilean healthcare institutions for CMFt during a defined study period. Eligible settings included emergency departments, trauma units, maxillofacial surgery services, and inpatient hospital care across public, private, and university-affiliated institutions, with no geographic restrictions within Chile. Studies of adults (≥18 years) were prioritized; studies including adolescents or mixed age groups were eligible when extractable age-stratified data were available.

*Exposition:* CMFt was defined as traumatic injury involving the extraoral facial region and/or facial skeleton, including facial soft tissue injuries and facial bone fractures, based on clinical documentation and/or imaging and/or intraoperative findings. Dentoalveolar trauma and isolated intraoral soft-tissue injuries were excluded from the primary CMFt definition because they follow distinct epidemiologic and care pathways. In Chile, these conditions are frequently managed through outpatient dental and primary-care services, including Explicit Health Guarantees (GES) for dental emergency care, rather than through hospital trauma admission pathways. CMFt had to be established by at least one of the following: (i) clinical assessment documented in medical records; (ii) imaging confirmation (radiography and/or CT); (iii) intraoperative findings.

- *Outcomes:* Two outcomes were prespecified, as defined below.
- *Prevalence of CMFt:* Proportion of CMFt cases among all patients within a defined healthcare denominator and time window. Prevalence estimates required a clearly defined denominator (e.g., all trauma attendances, all facial injury attendances, or all hospitalizations).
- *Patterns of CMFt:* Distribution of injury characteristics among CMFt cases, including anatomical site(s), injury type, etiology/mechanism, and key demographics.
- *Study Design:* We included observational designs (cross-sectional studies and prospective or retrospective cohorts, including registry-based studies). We excluded case reports, small case series without a denominator, forensic-only samples, and non-healthcare-attendance populations (e.g., autopsy studies). Studies reporting injuries solely as number of fractures (rather than affected patients) were included only when the unit of analysis was explicit and interpretable. Studies restricted to CMFt cases were eligible for pattern analyses but were not used for prevalence estimation due to the lack of an appropriate denominator.

### 2.3. Information Sources and Selection Process

The literature search followed PRISMA-S [33] and was conducted independently by two researchers (GSR, LSR). Six databases were included: EBSCOhost (Dentistry and Oral Sciences Source), LiLACS, PubMed, SCIelo, Scopus, and Web of Science. Studies published up to 5 February 2026, were considered, with no limits on the initial publication date, to encompass the entire body of the relevant literature on the topic. Search strategies were developed by an experienced researcher (GSR) using a PubMed thesaurus (MeSH terms) related to the topic, then adapted for the remaining databases (Supplementary File S1: Supplementary Table S1). Additionally, we contacted the authors of key studies to inquire about other relevant or ongoing research.

The resulting references were imported into EndNote™ (v21.5, The EndNote Team, Clarivate, Philadelphia, PA, USA, 2024) and duplicates were removed. Subsequently, full records were submitted to ASReview (v.2.1, ASReview LAB developers, Utrecht University, Utrecht, The Netherlands) [34], where two reviewers (GSR, PCG) confirmed the inclusion/exclusion of the entire set of references. This tool was used to support and expedite the reference screening process by applying active learning to prioritize articles most likely to meet the inclusion criteria, effectively ranking them according to their relevance. The identified articles were then uploaded in Rayyan (Rayyan Systems Inc., Cambridge, MA, USA) [35] for full-text reading. Additional studies cited in the reference lists of previously selected articles were identified using the same search terms across databases and registries, and their eligibility for inclusion in this SR was assessed. Excluded records, along with the reasons for exclusion, are reported in the table “Characteristics of excluded studies” (Supplementary File S1: Supplementary Table S2). Cohen’s kappa coefficient was calculated to evaluate inter-rater reliability during the screening and eligibility stages. Any discrepancies in study selection were resolved through consensus or adjudication by a third reviewer (ELF).

### 2.4. Data Collection Process

One reviewer (GSR) conducted data extraction using a standardized spreadsheet (Microsoft Excel 2024, Microsoft Corporation, Redmond, WA, USA). A second reviewer (PCG) independently checked a random 20% sample. When discrepancies were detected, data extraction was repeated and resolved by consensus. As the number of eligible studies was <30, both reviewers subsequently performed independent parallel extraction for all included articles to ensure accuracy and consistency. Based on the Cochrane Collaboration’s [36] guidance on study characteristics, we extracted information on study design, setting, population, CMFt definition and ascertainment, etiology/mechanism, and outcome data, including prevalence denominators when available and distributional data for anatomical site, injury type, etiology, and demographics. The full extraction framework is provided in Supplementary File S1. For studies with missing or unclear information, corresponding authors were contacted by email (two attempts, one week apart). In the absence of a response, the data were recorded as “not reported”.

### 2.5. Risk of Bias Assessment

A panel of two reviewers (GSR and PCG) assessed the risk of bias using the JBI critical appraisal tool for SR addressing prevalence questions [37]. This instrument evaluates key aspects of internal and external validity, including sample representativeness and adequacy, recruitment methods, the description and reporting of study participants and settings, data coverage of the identified sample, measurement of the condition of interest, the appropriateness of the statistical analyses, and the identification of confounding factors, subgroups, or differences.

### 2.6. Data Synthesis

A qualitative (narrative) synthesis was undertaken for each included study. Quantitative synthesis was conducted using a random-effects meta-analysis of single proportions to estimate pooled fracture-site-specific proportions across studies. Proportions were variance-stabilized using the Freeman-Tukey double arcsine transformation. Between-study heterogeneity was accounted for with a random-effects model, with τ^2^ estimated via restricted maximum likelihood (REML). Confidence intervals for pooled estimates were derived using the Hartung-Knapp-Sidik-Jonkman method, while 95% confidence intervals for individual studies were calculated using the Clopper–Pearson exact method. Statistical heterogeneity was assessed using Cochran’s Q test (*p* < 0.05) and quantified with the I^2^ statistic. Potential publication bias was evaluated for each outcome through visual inspection of funnel plot asymmetry and, where feasible, formally tested using Egger’s regression test (*p* < 0.05). Statistical analysis was performed using the “meta” package in R version 4.4.2 (R Foundation for Statistical Computing, Vienna, Austria; GSR).

Certainty of the evidence was evaluated using the GRADE (Grading of Recommendations, Assessment, Development and Evaluation) approach [38], which considers risk of bias, imprecision, inconsistency, indirectness, and publication bias to inform the summary of findings table (GSR, PCG). The certainty of evidence was categorized as high, moderate, low, or very low, starting at high and downgraded based on limitations identified across these domains. Although formal guidance for applying GRADE to systematic reviews of prevalence is limited, we followed the framework for baseline risk and overall prognosis [39], as recommended by Borges Migliavaca et al. [40].

### 2.7. Nationwide Administrative Database Study (DEIS)

In addition to the systematic review, a nationwide population-based observational study was conducted using administrative hospital discharge data from the Chilean Department of Health Statistics and Information (Departamento de Estadísticas e Información de Salud, DEIS), Ministry of Health. This complementary analysis aimed to characterize the temporal trends, etiological patterns, and hospital burden of CMFt at the national level, thereby addressing limitations related to sample size, geographic coverage, and temporal scope inherent to single-center studies.

The DEIS database includes mandatory records of all hospital discharges from public and private healthcare institutions in Chile and contains anonymized information on patient demographics, diagnoses coded according to the International Classification of Diseases, 10th Revision (ICD-10), length of hospital stay, discharge status, and external causes of injury when available. CMFt cases were defined as discharges with ICD-10 codes indicating facial soft tissue injuries or facial bone fractures. For the present study, all hospital discharges between January 2001 and December 2024 with a primary diagnosis corresponding to traumatic injuries (ICD-10 codes S00–S99 and T00–T14) were identified. Dentoalveolar trauma and isolated intraoral injuries were excluded to ensure consistency with the systematic review definitions.

Annual counts, proportions, and rates were calculated to describe national trends in CMFt admissions. Etiology was classified using ICD-10 external cause codes and grouped into clinically meaningful categories. Hospital burden was assessed using length-of-stay-based metrics, including mean length of stay and the proportion of admissions with prolonged hospitalization. Temporal trends were evaluated using ITS analyses [29]. Detailed variable definitions, coding algorithms, and statistical modeling procedures are provided in the Supplementary File S2. An interactive version of the data analysis is available in Spanish in: https://pa6t2w-gustavo-s0enz.shinyapps.io/cmftchile/ (accessed on 24 June 2026).

## 3. Results

A total of 167 articles were retrieved through database and registry searches. Handsearching and author contact were performed; no additional eligible studies were identified (Supplementary File S1: Supplementary Figure S1). After removing duplicates (n  =  81, 49%), 86 articles were screened by title–abstract–keyword reading, leaving 36 potentially eligible reports (Cohen’s kappa  =  0.93) and 1 article that could not be retrieved for full-text reading. After full-text reading, 19 articles were included (100% agreement). The reasons for and sources of the exclusion of 17 articles are detailed in Supplementary File S1: Table S2.

### 3.1. Characteristics of the Included Studies

Table S3 (Supplementary File S1) summarizes the characteristics of the included studies. A total of 19 studies on 20 hospital-based observational cohorts conducted in Chile were included, comprising three pediatric studies [10,11,12] and seventeen adult studies [11,13,14,15,16,17,18,19,20,21,22,23,24,25,26,27,28], and one article contributed separate pediatric and adult strata [11]. Article were published between 1999 and 2025. Most investigations were conducted in public tertiary or regional hospitals, particularly in the Metropolitan Region (Santiago) [10,14,17,18,20,22,23,24,26,27,28]. Four studies originated from the Valparaíso Region, [12,13,16,27] and two from central–southern regions [15,25]. Southern Chile was represented by Temuco [11] and Valdivia [21], and the northern region by one study from La Serena [19]. Two studies were conducted in a private occupational trauma center in Santiago [18,22], and another was based on records from a regional forensic medical service in Curicó [15]. Study periods ranged from 1990 to 2022. The duration of observation per study varied from less than one year to 10 years, with a mean duration of 3.9 years. The majority employed retrospective observational design, while only one study [27] used a prospective multicenter emergency registry.

Across the adult population, males predominated in all included studies, ranging from 63.3% to 91.5% of patients, whereas female representation ranged from 8.5% to 36.7%. Mean or median age in adult cohorts ranged from 27.4 to 42 years, with most injuries occurring in individuals between the second and fourth decades of life. In pediatric studies, male predominance was less pronounced, with male proportions ranging from 33.6% to 62.3%, and mean or grouped ages concentrated in children under 6 years of age.

Regarding etiology, interpersonal violence or assaults were the most frequently reported cause in adult populations, accounting for approximately 23.6% to 72.1% of cases when reported [16,20,21,22,27,28]. Traffic-related accidents represented 11.3% to 46.1%, while falls accounted for 7.0% to 27.0% of injuries across adult studies. Pediatric studies showed a different pattern, with falls and domestic or school-related accidents as the predominant causes [10,11,12]. Several studies reported a relevant proportion of other or non-specified causes, ranging from 7.1% to 33.3%, and in some cohorts, etiology could not be retrieved from clinical records [14,17].

Concerning anatomical distribution, mandibular fractures were the most frequently reported fracture type in adult surgical series, particularly in mandibular-only cohorts, [13,22,26,28], but also in mixed facial fracture studies [14,16,18,20,21,25]. Regarding this, mandibular angle, body, and condylar regions were reported as the most frequently affected sites. Zygomatic and orbitozygomatic fractures, followed by orbital and maxillary fractures, were commonly reported in broader maxillofacial trauma cohorts [16,17,19,23,24,27]. Pediatric fracture patterns were dominated by nasal and orbital fractures [10,11,12]. Only one study explicitly compared pre-pandemic and pandemic periods, reporting a reduction in total fracture volume during the pandemic but a persistent predominance of mandibular and zygomatic fractures across both periods [24].

### 3.2. Risk of Bias Assessment

Findings are summarized in Supplementary File S1: Supplementary Table S4. Overall, the evidence base was mainly limited by unclear source populations and referral pathways, incomplete reporting of case capture and sample coverage, lack of sample-size or precision justification, and insufficiently specified diagnostic/coding criteria and measurement procedures. Although all studies adequately described their participants and settings, most did not report uncertainty estimates or clearly address missing data, leading to concerns regarding the appropriateness of statistical reporting. The response-rate domain was considered not applicable because the studies included were predominantly retrospective and record-based.

### 3.3. Evidence Synthesis and Certainty of the Evidence

Pediatric CMFt accounted for 2.6% of all emergency consultations among children ≤15 years, whereas in a mixed-age emergency cohort, CMFt represented 6.1% of consultations [10,11]. Mandibular fractures were the most frequent, with a pooled proportion of 45.3% (95% CI: 31.0–59.9), followed by zygomatic fractures at 24.2% (95% CI: 18.6–30.4). The pooled proportion of nasal fractures was 16.3% (95% CI: 0.1–49.1), while orbital fractures accounted for 7.6% (95% CI: 3.3–13.5) and maxillary fractures for 8.6% (95% CI: 5.2–12.8). Less frequent patterns included NOE fractures (6.1%; 95% CI: 0.0–24.0), Le Fort fractures (4.3%; 95% CI: 1.6–8.2), panfacial fractures (4.2%; 95% CI: 0.5–11.0), and frontal fractures (2.6%; 95% CI: 0.0–9.5). Substantial heterogeneity was present in all the estimates (I^2^ > 80%, *p* < 0.05) (Supplementary File S1: Supplementary Figure S2). Regarding publication bias (Supplementary File S1: Supplementary Figure S3), funnel plots were largely symmetric and Egger’s regression tests (where estimable) did not indicate small-study effects, suggesting no clear evidence of its presence. Overall certainty ranged from low to very low, mainly downgraded for risk of bias (observational design and potential selection/convenience sampling) and inconsistency (between-study heterogeneity and non-overlapped confidence intervals), with additional downgrading for imprecision in outcomes with wide confidence intervals. Certainty was rated low for frontal, orbital, zygomatic, maxillary, Le Fort, and panfacial fractures, and very low for nasal, mandibular, and NOE fractures (Supplementary File S1: Supplementary Table S5).

### 3.4. Temporal Trends According to the Nationwide Administrative Database Study (DEIS)

**Figure 1:**
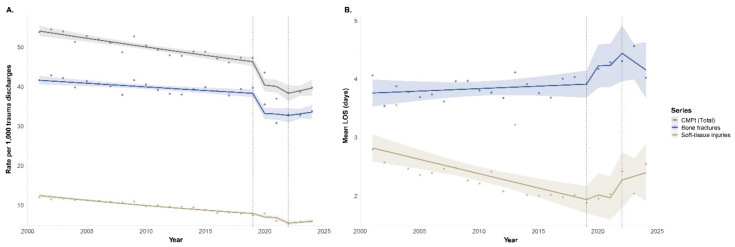
National CMFt admission rates and mean length of stay over time (DEIS, 2001–2024). (**A**) Annual CMFt admission rates per 1000 trauma discharges, shown for total CMFt and stratified by bone fractures and soft-tissue injuries. Points indicate observed annual rates; solid lines show segmented (interrupted time-series) fitted values with shaded 95% confidence bands. Vertical dotted lines denote the pre-specified interruption periods (COVID-era and post-2022). (**B**) Annual mean length of stay (LOS, days) for CMFt total, bone fractures, and soft-tissue injuries, with fitted segmented trends and 95% confidence bands.

Across 2001–2024, CMFt rates per 1000 trauma discharges declined, with an additional COVID-period level reduction and a post-2022 partial rebound. In ITS negative binomial models, the baseline (2001) CMFt rate was 54.2/1000 (95% CI 53.3–55.2) with a pre-2020 decline (IRR/year 0.991, 95% CI 0.990–0.993), a 2020–2021 level decrease (IRR 0.880, 95% CI 0.779–0.995), a further post-2022 level decrease (IRR 0.850, 95% CI 0.830–0.869), and a post-2022 slope increase (IRR 1.026, 95% CI 1.020–1.032) (Supplementary File S3: Supplementary Table S1). Bone fractures predominated (baseline 41.8/1000, 95% CI 40.8–42.8) and showed similar dynamics (IRR/year 0.995, 95% CI 0.993–0.998; COVID level IRR 0.871, 95% CI 0.781–0.973; post-2022 level IRR 0.866, 95% CI 0.838–0.895; post-2022 slope IRR 1.017, 95% CI 1.009–1.026). Soft-tissue CMFt was less frequent (baseline 12.6/1000, 95% CI 12.2–13.0), declined more steeply pre-2020 (IRR/year 0.975, 95% CI 0.972–0.979), and showed no clear COVID level effect (IRR 0.913, 95% CI 0.753–1.108), but had a marked post-2022 level reduction (IRR 0.751, 95% CI 0.713–0.790) and slope increase (IRR 1.069, 95% CI 1.059–1.078) (Supplementary File S3: Supplementary Tables S2 and S3) (Figure 1A). Across age groups and sex strata, CMFt rates per 1000 trauma discharges are higher in males, with a sharp rise in ages 0–9 years old, but secular declines in older groups, plus a 2020–2021 drop and partial post-2022 rebound (Supplementary File S3: Supplementary Figure S1).

**Figure 2:**
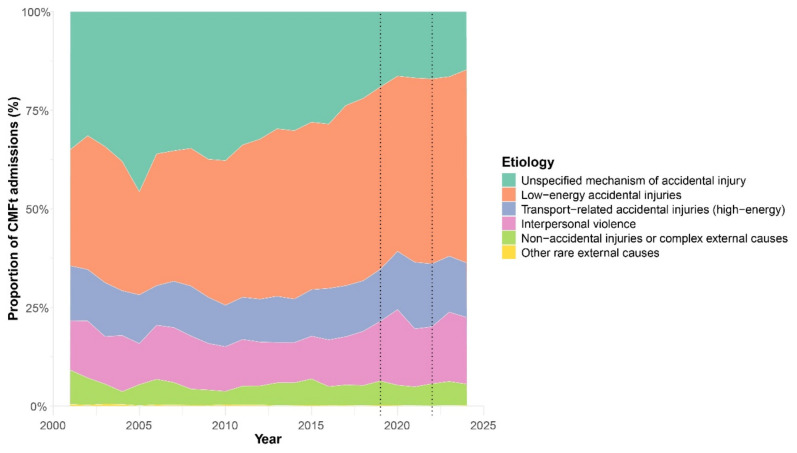
Temporal composition of CMFt admissions by etiology group (DEIS, 2001–2024). Vertical dotted lines denote the pre-specified interruption periods (COVID-era and post-2022).

Etiology shifted toward low-energy accidental injuries, with a concurrent rise in interpersonal violence, while transport-related high-energy injuries remained comparatively stable (Figure 2). Etiologic composition shows age- and sex-related trends, where low-energy accidental mechanisms increasingly dominated pediatric and older-adult admissions, whereas the rise in interpersonal violence was concentrated in adolescents and working-age adults and was more pronounced among males. Transport-related high-energy injuries were comparatively more prominent in younger strata and otherwise remained relatively stable (Supplementary File S3: Supplementary Figure S2).

**Figure 3:**
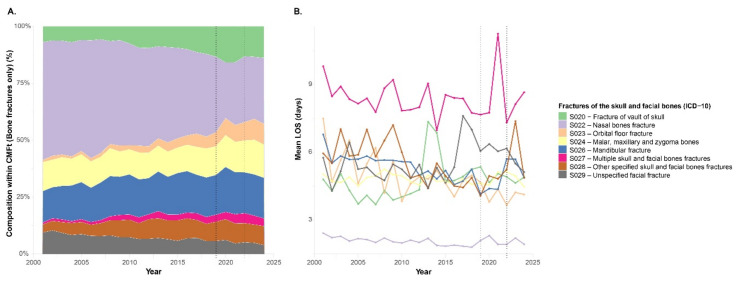
Bone-fracture case-mix and diagnosis-specific LOS trends (DEIS, 2001–2024). (**A**) Annual composition of CMFt admissions restricted to bone fractures, by ICD-10 diagnosis group (S020-S029), expressed as percentage of all bone-fracture CMFt admissions. Vertical dotted lines denote the pre-specified interruption periods (COVID-era and post-2022). (**B**) Annual mean LOS (days) by ICD-10 fracture diagnosis group (S020–S029). Vertical dotted lines denote the pre-specified interruption periods (COVID-era and post-2022).

Among fractures, nasal bone fractures (S022) remained most frequent but decreased in share, alongside increases in mandibular (S026) and late-series rises in orbital floor (S023) and skull vault fractures (S020); S029 decreased over time (Figure 3A). S022 remained the most frequent diagnosis across age–sex strata, but their relative share declined over time as S026 and midface fractures increased. Pediatric strata also showed a rising contribution of S020 (Supplementary File S3: Supplementary Figure S3). Low-energy accidental injuries predominated across ICD-10 fracture types, S022 showed the highest share of unspecified accidental mechanisms, interpersonal violence contributed relatively more to midface/mandibular fractures (S023/S024/S026), and transport-related high-energy injuries peaked in multiple skull and facial bone fractures (S027) (Supplementary File S3: Supplementary Figure S4).

Baseline mean LOS was 2.83 days for soft-tissue CMFt, with fractures +0.94 days longer; soft-tissue LOS declined pre-2020 (−0.049 days/year), with greater variability around 2020–2022 (Supplementary File S3: Supplementary Table S4) (Figure 1B). Mean LOS exhibited a strong age trend, remaining low and stable in children/adolescents but increasing in older adults, with fracture admissions consistently exceeding soft-tissue CMFt. In addition, several strata show transient perturbations around 2020–2022 with partial normalization thereafter (Supplementary File S3: Supplementary Figure S5). The odds of prolonged LOS (≥5 days) decreased pre-2020 (OR/year 0.992, 95% CI 0.986–0.998), increased in 2020–2021 (OR 1.153, 95% CI 1.082–1.229) and post-2022 (OR 1.216, 95% CI 1.126–1.314), then declined after 2022 (OR/year 0.968, 95% CI 0.944–0.992) (Supplementary File S3: Supplementary Table S5 and Supplementary Figure S6). Mean LOS was highest for transport-related high-energy injuries and interpersonal violence, and lower for low-energy/unspecified accidental mechanisms (Supplementary File S3: Supplementary Figure S7). This pattern is mirrored by diagnosis-specific trends, where multiple skull and facial bone fractures (S027) show the longest mean LOS over time (8.35 days), whereas isolated nasal bone fractures (S022) exhibit the shortest and most stable LOS (2.08 days) (Supplementary File S3: Supplementary Table S6) (Figure 3B).

## 4. Discussion

This study combines an SR/MA of hospital-based CMFt evidence with a nationwide ITS analysis of administrative discharge data, providing a dual-synthesis framework for settings without dedicated CMFt registries. In Chilean hospital cohorts, CMFt predominantly affected males, interpersonal violence was the most frequently reported adult etiology, and mandibular and zygomatic fractures represented the largest pooled fracture-site proportions. These findings are aligned with international evidence [7,41,42,43]. However, denominator-based prevalence was rarely reported, and substantial heterogeneity indicates that pooled estimates should be interpreted as central tendencies across heterogeneous hospital series rather than precise national parameters. The DEIS analysis complemented these findings by quantifying national admission trends and inpatient burden. CMFt admission rates declined over the study period, showed a COVID-period disruption, and partially rebounded after 2022. Resource use was mainly fracture-driven, with fractures associated with longer LOS compared to soft-tissue injuries and with greater hospitalization burden in more complex diagnoses and high-energy or interpersonal-violence mechanisms.

### 4.1. Strengths and Limitations of the Study

The main strength of this study is its integration of two complementary evidence sources: hospital-based observational studies and nationwide administrative discharge data. This design adds system-level context to fragmented single-center reports and provides a reproducible analytical template for countries without dedicated CMFt registries.

Limitations are mainly related to the underlying evidence base and the nature of administrative data. Most included studies were single-center observational series with heterogeneous or undefined denominators, unclear referral pathways, incomplete reporting of case capture, and selected case mixes, particularly surgically treated cohorts. Although study-level moderators may explain part of the between-study variability, subgroup analyses were underpowered. These features may under-represent injuries commonly managed in ambulatory settings and limit the national representativeness of pooled estimates. DEIS provides nationwide coverage and long-term trends but captures hospital discharges rather than unique individuals, depends on ICD-10 coding quality, and does not capture the outpatient burden of CMFt or concomitant injuries recorded outside the primary diagnosis. Consequently, our estimates should be interpreted as describing hospital-based CMFt involving facial soft tissues and/or facial bones, rather than the total burden of orofacial trauma, which would require linkage with outpatient dental and primary-care datasets.

### 4.2. Effects on Patient Care and Health Policy

Clinically, the predominance of mandibular and zygomatic fractures supports prioritizing care pathways for the early identification of occlusal, airway, and ocular compromise, timely CT-based assessment when indicated, and access to fixation resources [6]. At the service level, the longer LOS observed for fracture admissions highlights the value of optimizing time-to-imaging, time-to-decision, time-to-operating room, and discharge coordination. Etiologic patterns also support combining acute surgical preparedness [44] with injury-prevention strategies focused on interpersonal violence, transport-related injuries, and low-energy accidental mechanisms.

### 4.3. Future Research Directions

Future efforts should prioritize multicenter CMFt registries with fixed denominators, harmonized case definitions, standardized fracture classification, and explicit reporting of imaging confirmation [45], referral pathways, and treatment setting. Linkage between emergency department presentations, admissions, surgical procedures, and outpatient dental or primary-care records would allow for estimation of the full orofacial trauma burden and clarify how case selection shapes differences between hospital series and administrative discharge data. The dual-synthesis template used here can also be replicated in other countries with centralized discharge databases but no dedicated CMFt registry, enabling more comparable national profiles across settings [6].

## Data Availability

The data that support the findings of this study are available from the corresponding author, G.S.-R., upon reasonable request.

## Supplementary Materials

The following supporting information can be downloaded at: https://www.mdpi.com/article/10.3390/cmtr19030032/s1, Supplementary File S1: Table S1: Search queries used in the systematic review; Table S2: Characteristics of the excluded studies after full-text reading; Table S3: Characteristics of the included studies; Table S4: Risk of Bias assessment; Table S5: Summary of Findings table; Figure S1: PRISMA 2020 flow diagram for the systematic searching process; Figure S2: Forest plot of single-proportion random-effects meta-analysis of the prevalence of maxillofacial trauma in Chile. CI: Confidence Interval, IV: Inverse Variance; Figure S3: Funnel plot of single-proportion random-effects meta-analysis of the prevalence of maxillofacial trauma in Chile. Supplementary File S2: Table S1: ICD-10 codes related to CMFt; Supplementary methods for the nationwide administrative database study using DEIS hospital discharge data. Supplementary File S3: Table S1: Interrupted time series (negative binomial) model for national CMFt rates within hospitalized trauma discharges; Table S2: Interrupted time series (negative binomial) model for national CMFt rates within hospitalized trauma discharges (bone fractures); Table S3: Interrupted time series (negative binomial) model for national CMFt soft-tissue injury rates within hospitalized trauma discharges; Table S4: Segmented linear regression (interrupted time series) with injury-type interactions for mean length of hospital stay (LOS, days) after CMFt, 2001–2024; Table S5: Interrupted time series (logit-linear segmented regression) for the proportion of bone fractures admissions with prolonged hospitalization (LOS ≥ 5 days), 2001–2024; Table S6: Mean LOS according to the type of bone fracture, 2001–2024; Figure S1: Trends in rates according to age groups and sex (ITS); Figure S2: Etiology composition within CMFt (bone fractures) according to age and sex; Figure S3: Diagnostic composition within CMF fractures by age group and sex, 2001–2024; Figure S4: Etiology composition within CMFt (bone fractures); Figure S5: Mean length of stay by age group and sex (CMFt admissions), 2001–2024; Figure S6: Proportion of prolonged hospitalization (LOS ≥ 5 days) after bone fractures within CMFt (ITS); Figure S7: Mean LOS of CMFt by etiology.

## Abbreviations

The following abbreviations are used in this manuscript:

CMFt	Craniomaxillofacial trauma
SR	Systematic review
MA	Meta-analysis
SR/MA	Systematic review and meta-analysis
ITS	Interrupted time-series
LOS	Length of stay
DEIS	Departamento de Estadísticas e Información de Salud
ICD-10	International Classification of Diseases, 10th Revision
JBI	Joanna Briggs Institute
PRISMA	Preferred Reporting Items for Systematic Reviews and Meta-Analyses
PRISMA-S	PRISMA extension for Reporting Literature Searches in Systematic Reviews
PROSPERO	International Prospective Register of Systematic Reviews
MeSH	Medical Subject Headings
GRADE	Grading of Recommendations, Assessment, Development and Evaluation
REML	Restricted maximum likelihood
CI	Confidence interval
CT	Computed tomography
NOE	Naso-orbito-ethmoidal
