# Supplementary material for: Epidemiology of Craniomaxillofacial Trauma in Chile: A Systematic Review and 24-Year Nationwide Interrupted Time-Series Analysis
Source: Craniomaxillofac Trauma Reconstr. 2026 Jul 3;19(3):32. doi: 10.3390/cmtr19030032 (PMC13398010; doi:10.3390/cmtr19030032)
Supplement: Supplementary file 1 [file cmtr-19-00032-s001.zip › Supplementary File S1.pdf]

Data collection process: Data extraction framework

- *Study characteristics*: location (city/region when reported), institution type (public/private/university-affiliated), setting (emergency department/trauma unit/maxillofacial service/inpatient), design (cross-sectional; retrospective/prospective cohort; registry).
- *Participants*: sample size; age summary (mean±SD, median [IQR], or range); sex distribution; socioeconomic/education variables when reported (as defined by each study).
- *Clinical context*: polytrauma or relevant comorbidities/risk context when reported (e.g., alcohol/substance involvement, chronic conditions), recorded as reported by the original study.
- *CMFt definition and ascertainment*: operational definition (anatomic scope), diagnostic approach (clinical/imaging/intraoperative), and any classification system used.
- *Etiology/mechanism*: categorized as reported (e.g., interpersonal violence, road traffic, falls, sports, occupational), including alcohol/substance involvement when available.
- *Outcome data*: prevalence numerator/denominator (when applicable) and distributional data for pattern outcomes (anatomic site, injury type, etiology, demographics).

Supplementary Table S1. Search queries used in the systematic review

Database	Search query	Filters	Results
**EBSCOhost**	(prevalen* OR incidence OR epidemiolog* OR descript* OR character* OR trend* OR prevalencia OR incidencia OR epidemiolog* OR descript* OR caracter* OR tendencia*) AND ("maxillofacial trauma" OR "facial trauma" OR "facial injuries" OR "maxillofacial injuries" OR "facial fracture*" OR "orbital fracture*" OR "zygomatic fracture*" OR "jaw fracture*" OR "mandibular fracture*" OR "maxillary fracture*" OR "mandibular injur*" OR "trauma maxilofacial" OR "trauma facial" OR "lesiones maxilofaciales" OR "fractura* facial*" OR "fractura* mandibular*" OR "fractura* maxilar*" OR "fractura* orbitaria*" OR "fractura* cigomatica*") AND (Chile* OR Chilean)	None	36
**LiLACS (Full collection)**	(prevalen* OR incidence OR epidemiolog* OR descript* OR character* OR trend* OR prevalencia OR incidencia OR epidemiolog* OR descript* OR caracter* OR tendencia*) AND ("maxillofacial trauma" OR "facial trauma" OR "facial injuries" OR "maxillofacial injuries" OR "facial fracture*" OR "orbital fracture*" OR "zygomatic fracture*" OR "jaw fracture*" OR "mandibular fracture*" OR "maxillary fracture*" OR "mandibular injur*" OR "trauma maxilofacial" OR "trauma facial" OR "lesiones maxilofaciales" OR "fractura* facial*" OR "fractura* mandibular*" OR "fractura* maxilar*" OR "fractura* orbitaria*" OR "fractura* cigomatica*") AND (Chile* OR Chilean)	None	36
**PubMED**	("Prevalence"[Mesh] OR charact* OR descript* OR epidemiolog* OR incidence OR trend*) AND (("Facial Injuries"[Mesh] OR "Maxillofacial Injuries"[Mesh] OR "Orbital Fractures"[Mesh] OR "Zygomatic Fractures"[Mesh] OR "Jaw Fractures"[Mesh] OR "Maxillary Fractures"[Mesh] OR "Mandibular Fractures"[Mesh] OR "Mandibular Injuries"[Mesh]) OR (("Facial Bones"[Mesh] OR "Frontal Bone"[Mesh] OR "Maxilla"[Mesh] OR "Mandible"[Mesh] OR "Jaw"[Mesh] OR "Nasal Bone"[Mesh] OR "Zygoma"[Mesh] OR "Orbit"[Mesh] OR "Ethmoid Bone"[Mesh]) AND ("Wounds and Injuries"[Mesh] OR "Fractures, Bone"[Mesh]))) AND (Chile*)	None	25
**SciELO**	(prevalen* OR incidence OR epidemiolog* OR descript* OR character* OR trend* OR prevalencia OR incidencia OR epidemiolog* OR descript* OR caracter* OR tendencia*) AND ("maxillofacial trauma" OR "facial trauma" OR "facial injuries" OR "maxillofacial injuries" OR "facial fracture*" OR "orbital fracture*" OR "zygomatic fracture*" OR "jaw fracture*" OR "mandibular fracture*" OR "maxillary fracture*" OR "mandibular injur*" OR "trauma maxilofacial" OR "trauma facial" OR "lesiones maxilofaciales" OR "fractura* facial*" OR "fractura* mandibular*" OR "fractura* maxilar*" OR "fractura* orbitaria*" OR "fractura* cigomatica*") AND (Chile* OR Chilean)	None	22
**Scopus**	TITLE-ABS-KEY ((prevalen* OR incidence OR epidemiolog* OR descript* OR character* OR trend* OR prevalencia OR incidencia OR epidemiolog* OR descript* OR caracter* OR tendencia*) AND ("maxillofacial trauma" OR "facial trauma" OR "facial injuries" OR "maxillofacial injuries" OR "facial fracture*" OR "orbital fracture*" OR "zygomatic fracture*" OR "jaw fracture*" OR "mandibular fracture*" OR "maxillary fracture*" OR "mandibular injur*" OR "trauma maxilofacial" OR "trauma facial" OR "lesiones maxilofaciales" OR "fractura* facial*" OR "fractura* mandibular*" OR "fractura* maxilar*" OR "fractura* orbitaria*" OR "fractura* cigomatica*") AND (Chile* OR Chilean))	None	20
**Web of Science**	(prevalen* OR incidence OR epidemiolog* OR descript* OR character* OR trend* OR prevalencia OR incidencia OR epidemiolog* OR descript* OR caracter* OR tendencia*) (All Fields) and ("maxillofacial trauma" OR "facial trauma" OR "facial injuries" OR "maxillofacial injuries" OR "facial fracture*" OR "orbital fracture*" OR "zygomatic fracture*" OR "jaw fracture*" OR "mandibular fracture*" OR "maxillary fracture*" OR "mandibular injur*" OR "trauma maxilofacial" OR "trauma facial" OR "lesiones maxilofaciales" OR "fractura* facial*" OR "fractura* mandibular*" OR "fractura* maxilar*" OR "fractura* orbitaria*" OR "fractura* cigomatica*") (All Fields) and (Chile* OR Chilean) (All Fields)	None	28

Supplementary Table S2. Characteristics of the excluded studies after full-text reading

Author	Wrong				Notes
	P	E	C	O	
Andrades et al. 2012 [1]		●			Management of intractable bleeding
Andrades et al. 2023 [2]		●			Post-operative complications from orbital reconstruction
Briones et al. 2003 [3]		●			Post-operative complications from mandibular fractures
la Fuente et al. 2023 [4]		●			Post-operative complications from mandibular fractures
Gonzalez et al. 2015 [5]				●	Sample already partially-contained in another included study
Gonzalez et al. 2015 [6]		●			Surgical technique - orbital reconstruction
Lagos et al. 2025 [7]	●				Only female individuals included
Muñoz et al. 2011 [8]					Abstract only (not retrived)
Nasi et al. 2024 [9]		●			Post-operative complications from mandibular fractures
Olate et al. 2011 [10]	●				Cohort from Brazil
Ormeño et al. 2022 [11]		●			Unspecific diagnosis – “ocular and orbit trauma”
Pedemonte et al. 2020 [12]		●			Surgical technique - orbital reconstruction
Pérez et al. 2015 [13]	●				Sample included in a more recent included study
Rivera 2017 [14]				●	Does not report prevalence
Sandoval et al. 2017 [15]		●			Includes maxillofacial infections
Silva 2023 [16]				●	Individual reports from Dictatorship period in Chile
Werlinger et al. 2023 [17]	●	●			Analysis over a subset of an included study, with unspecified diagnosis
Werlinger et al. 2025 [18]	●				Subset of an included study only describing older adults

P: Population, E: Exposition, C: Comparator, O: Outcome

References

1. Andrades C P, Román F JL, Bartel A R, Borel B C, Hernández N R, Rojas S R, Lankin B J, Villalobos A R: Hemorragia incoercible por trauma maxilofacial: experiencia del Hospital del Trabajador. Revista chilena de cirugía 64: 169, 2012.
2. Andrades Cvitanic P, Grass Briones B, Zang J, Rios Vergara M, Rodriguez Schneider D, Borel Baeza C: Complicaciones postoperatorias en fractura de órbita: experiencia en el Hospital del Trabajador de Santiago. Rev Cir (Mex) 75, 2023.
3. Briones D, Herbozo P, Gomes IL: Elección de tratamiento y complicaciones asociados a diferentes tratamientos de fracturas mandibulares. Medicina Oral (16656024) 5: 84, 2003.
4. la Fuente Escalona M de, Moris Vidal HF, Salas Martínez M, Gunckel Muñoz R, Lazo Peréz D, Mardones Muñoz M: Retiro De Elementos De Osteosíntesis Asociadas A Cirugías De Trauma Maxilofacial En Hospital Público De Santiago De Chile. Estudio Retrospectivo De 4 Años. Revista Española de Cirugía Oral y Maxilofacial 45, 2023.
5. Gonzalez Mora LE, Vargas Farren I, Pedemonte Trewhela C, Canales Trkovic M, Sáez Salas F, Verdugo-Avello F: Análisis de las Fracturas Mandibulares Causadas por Accidentes Laborales: Estudio Descriptivo Retrospectivo. International journal of odontostomatology 9: 198, 2015.
6. González M E, Pedemonte T C, Vargas F I, Verdugo-Avello F: EVALUACIÓN CLÍNICA DE LA RECONSTRUCCIÓN ORBITARIA POST TRAUMÁTICA MEDIANTE MALLAS DE TITANIO. Revista chilena de cirugía 67: 252, 2015.
7. Lagos Tissie D, Faúndes Pinto M, Silva Melo J, Montoya Barrena A, Contreras Vicencio C: Lesionología maxilofacial en violencia de género: Estudio descriptivo de casos de Valparaíso, Chile. Revista Española de Medicina Legal 51: 500458, 2025.
8. Muñoz MM, Gomez EN, Ahumada RB: Facial trauma prevalence in the north area of Santiago. Int J Oral Maxillofac Surg 40: 1025, 2011.
9. Nasi Toso M, Diaz Sotomayor F, Sanino Zavala I, Diaz González J, Quitral Argandoña R, Olivares Unamuno I: Prevalence of postoperative complications of mandibular fractures at the Dr. Gustavo Fricke Hospital, Chile: a six-year study. Journal of Oral Research 13: 409, 2024.
10. Olate S, Lima SM, Sawazaki R, Moreira RWF, Moraes M de: Variables Related to Surgical and Nonsurgical Treatment of Zygomatic Complex Fracture. Journal of Craniofacial Surgery 22: 1200, 2011.
11. Ormeño Illanes J: Epidemiology and trends of ocular trauma hospitalizations in Chile from 2001 to 2020. J Fr Ophtalmol 45: 1055, 2022.
12. Pedemonte Trewhela C, Carmona Avendaño AP, González Mora E, Vargas Farren I, Huentequeo Molina C, Noguera Pantoja A: Abordaje Transconjuntival: Primera Elección en Trauma Orbitario. Revista Española de Cirugía Oral y Maxilofacial 42, 2020.
13. Pérez Gutiérrez H, Donoso Hofer T, Mardones Muñoz M, Bravo Ahumada R: Epidemiología de Tratamientos Quirúrgicos Maxilofaciales en un Hospital Público en Santiago de Chile: Estudio Retrospectivo de 5 Años. International journal of odontostomatology 9: 37, 2015.
14. Rivera P. H: Traumatismos máxilo faciales en el niño. ARS MEDICA Revista de Ciencias Médicas 18: 43, 2017.
15. Sandoval Tobar ME, Reyes Court D, Sanhueza Olea V: Epidemiología de la patología quirúrgica que afecta al territorio maxilofacial, tratada bajo anestesia general en el Hospital de Urgencia Asistencia Pública entre 2014 y 2016. Revista Chilena de Cirugía 69: 289, 2017.
16. Silva J: Oral and maxillo-facial injuries in victims of political repression during the Chilean dictatorship. Torture Journal 33: 23, 2023.
17. Werlinger F, Villalón M, Duarte V, Sepúlveda P: Interpersonal Violence and Maxillofacial Injuries: Toward an Active Surveillance Proposal Through the Presentation Profile in Hospital Emergency Services. Violence Vict 38: 787, 2023.
18. Werlinger F, Villalón M, Duarte V: Falls and Interpersonal Violence, the Main Patterns of Maxillofacial Trauma in Older Adults in Chile. Gerodontology 42: 554, 2025.

Supplementary Table S3. Characteristics of the included studies

Study	Location/Setting/Study design (period)	Patients evaluated	Age	Sex	Etiology	Type of trauma
**Pediatric population**						
**Duarte 1999 [12]**	Valparaíso Region, Chile. Public hospitals (Hospital Dr. Gustavo Fricke, Hospital de Niños y Cunas de Viña del Mar, Hospital Carlos Van Buren) / Pediatric Emergency Units / Retrospective observational hospital-based study (January 1995 - December 1998).	213 hospitalized pediatric patients (<15 years) with maxillofacial injuries.	0-5 years (54%); 6-11 years (31%); 12-15 years (15%).	Male 56.3%; Female 43.7%.	Falls from height 30.0%; traffic accidents 18.8%; dog bites 14.1%; blunt trauma with objects 6.6%; sports injuries 6.6%; child abuse 3.8%; other causes 2.8%; not reported 17.4%.	Extraoral facial soft-tissue injuries (n=99). Facial fractures (n=114; dentoalveolar excluded): nasal (n=48); orbital (n=38); midface (n=11); mandibular (n=7); multiple facial bones (n=8).
**Collao-Gonzalez et al. 2014 [10]**	Santiago, Chile. Public pediatric hospital (Exequiel González Cortés Children’s Hospital) / Emergency Department / Retrospective observational study (May 2006 - April 2009)	293,090 pediatric emergency consultations; 7,617 patients with maxillofacial trauma (2.6%).	Mean 5.6 years; range 1 month-15 years. Age groups: 0-5 (56.3%); 6-12 (35.8%); 13-15 (7.7%).	Male 62.3%; Female 37.7%.	Falls 53.5%; domestic accidents 28.8%; animal bites 6.3%; interpersonal violence 3.9%; motor vehicle accidents 3.4%; other causes <3%.	Extraoral facial soft-tissue injuries (n=7,367). Facial fractures in 6% of trauma patients (n=496; one fracture per patient): nasal 96.4%; mandibular 1.8%; maxillary 0.6%; zygomatic 0.6%; orbital 0.6%.
**Medina et al. 2006 [11]**	Temuco, Chile. Public general hospital (Hospital Regional Hernán Henríquez Aravena) / Emergency Department / Retrospective descriptive study (April 2002 - March 2003)	742 patients (random sample from 34,931 emergency consultations); 9 pediatric patients with maxillofacial fractures (subset of 45 total fracture cases).	0-14 years.	Male 33.6%; Female 66.4%.	Falls 52.9%; school accidents 23.5%; assaults 5.9%; other causes / not reported 17.7%.	Facial bone fractures: frontal (n=1); naso-orbito-ethmoidal (NOE) (n=8).
**Adults**						
**Cuellar et al. 2019 [14]**	Santiago, Chile. Public tertiary emergency hospital (Hospital de Urgencia Asistencia Pública, HUAP) / Maxillofacial Surgery Unit / Retrospective observational study based on surgical protocols (December 2014 - December 2017).	127 adult patients surgically treated under general anesthesia; 259 maxillofacial fractures (nasal fractures and ambulatory cases excluded).	Mean 33 years (range 15-92). Age groups: 15-19 (11.8%); 20-29 (33.9%); 30-39 (22.0%); 40-49 (17.3%); 50-59 (11.0%); ≥60 (3.9%).	Male 85.8%; Female 14.2%.	Not reported.	Fracture distribution (n=255): frontal (n=1); zygomatic body (n=27); zygomatic arch (n=4); maxilla (n=13); Le Fort I (n=4); Le Fort II (n=2); orbital floor (n=4); mandibular fractures (n=200: symphysis 7, parasymphysis 58, body 39, angle 47, ramus 7, condyle 41); panfacial fractures (n=2).
**Espinosa et al. 2019 [15]**	Curicó, Chile. Public forensic medical service (Servicio Médico Legal, SML Curicó) / Retrospective observational descriptive	79 adult patients (≥17 years) with maxillofacial injuries.	≥17 years.	Male 63.3%; Female 36.7%.	Interpersonal violence 53.2%; domestic violence 8.9%; traffic accidents	Soft-tissue injuries in 49 patients. Fractures in 26 patients (29.6%): nasal (n=1); zygomatic (n=1); NOE (n=1); Le Fort III (n=1).

	study (May 2017 - May 2018).				43.0% (n=34).	
**Faille & Badillo, 2018 [16]**	Valparaíso, Chile. Public tertiary hospital (Hospital Carlos Van Buren) / Maxillofacial Surgery Unit / Retrospective observational study (January 2010 - December 2014)	414 surgically treated maxillofacial fractures.	Mean 34.04 years (range 2-74). Age groups: 0-9 (3.04%); 10-19 (11.74%); 20-29 (30.00%); 30-39 (22.17%); 40-49 (16.52%); 50-59 (9.57%); 60-69 (5.65%); 70-80 (1.30%).	Male 83%; Female 17%.	Interpersonal violence 47.33%; falls 25.45%; traffic accidents 19.6%; other causes 7.62%.	Medial orbital wall (n=4); orbitozygomatic (n=99); zygomatic arch (n=38); nasomaxillary (n=29); nasal (n=22); NOE (n=21); Le Fort I (n=23); Le Fort II (n=17); Le Fort III (n=10); mandibular (n=210).
**De la Fuente et al. 2023 [17]**	Santiago, Chile. Public hospital (Hospital San José) / Maxillofacial Surgery Unit / Retrospective study (2018-2021).	176 maxillofacial trauma surgeries.	Mean 36.63 years.	Male 86.36%; Female 13.64%.	Not reported.	Orbit (n=7); orbitozygomatic (n=42); NOE (n=1); zygomatic (n=7); Le Fort (n=6); mandible (n=105); panfacial (n=8).
**Gonzalez et al. 2015 [18]**	Santiago, Chile. Private trauma center (Hospital Clínico Mutual de Seguridad) / Emergency Department and Maxillofacial Surgery Unit / Retrospective descriptive observational study (2009-2011).	283 adult patients with maxillofacial fractures (nasal fractures excluded).	Mean 40.5 ± 20.5 years (range 18-76). Age groups: 10-19 (1.4%); 20-29 (21.9%); 30-39 (25.1%); 40-49 (25.4%); 50-59 (18.7%); ≥60 (7.4%).	Male 91.5%; Female 8.5%.	Traffic accidents 39.2%; interpersonal violence 23.6%; blunt trauma with objects 15.5%; falls 15.2%; industrial accidents 3.5%; other causes 2.8%.	Frontal (n=40); orbit (n=88); zygomatic (n=136); NOE (n=13); Le Fort (n=29); maxillary (n=25); mandibular (n=60); panfacial (n=12).
**Medina et al. 2006 [11]**	Temuco, Chile. Public general hospital (Hospital Regional Hernán Henríquez Aravena) / Emergency Department / Retrospective descriptive study (April 2002 - March 2003)	742 patients (random sample); 26 adult patients with maxillofacial fractures (subset of 45 total fracture cases).	≥15 years.	Male 55.9%; Female 44.1%.	Interpersonal violence 46.4%; traffic accidents 14.3%; falls 7.1%; unknown / not reported 25%.	Frontal (n=2); NOE (n=17); zygomatic (n=2); mandibular (n=6).
**Quitral et al. 2022 [13]**	Viña del Mar, Chile. Public tertiary hospital (Hospital Dr. Gustavo Fricke) / Maxillofacial Surgery Unit / Retrospective cross-sectional observational study (January 2014 - July 2020)	147 adult patients surgically treated for mandibular fractures.	Mean 40.8 years (range 2-81). Age groups: <20 (16.3%); 20-39 (49.7%); 40-59	Male 89.1%; Female 10.9%.	Interpersonal violence 47.6%; falls 19.7%; motor vehicle accidents 15.6%; firearm injury 2.7%; sports-related trauma 1.4%;	Mandibular fractures only (n=225): angle 27.1% (n=61); parasymphysis 24.0%; other mandibular sites (body, symphysis, condyle, ramus) 48.9%.

			(27.9%); ≥60 (6.1%).		other causes 3.4%; not reported 9.5%.	
**Raposo et al. 2013 [21]**	Valdivia, Chile. Public tertiary hospital (Hospital Base de Valdivia; referral public hospital for Región de Los Ríos) / Maxillofacial Surgery Department / Cross-sectional hospital-based retrospective review of surgically reduced fractures (January 2005-December 2010).	149 patients with maxillofacial trauma.	Mean 27.47 ± 10.9 years. Age groups: 0-9 (2.33%); 10-19 (22.09%); 20-29 (41.28%); 30-39 (14.53%); 40-49 (18.02%); 50-59 (1.74%).	Male 90.7%; Female 9.3%.	Interpersonal violence 45.9%; motor vehicle accidents 19.2%; falls 7.0%; other causes 19.2%; not reported 8.7%.	Distribution of 165 fractures: frontal (n=2); orbit (n=5); zygomatic (n=31); nasal (n=1); Le Fort I (n=1); Le Fort II (n=2); Le Fort III (n=2); mandibular (n=121).
**Raposo et al. 2021 [20]**	Santiago, Chile. Public tertiary hospital (Hospital Barros Luco Trudeau) / Maxillofacial Surgery Unit / Retrospective cross-sectional descriptive study during COVID-19 pandemic (March-August 2020).	25 patients with maxillofacial trauma.	≥17 years.	Not reported.	Interpersonal violence 64%; traffic accidents 20%; falls 8.7%.	Distribution of 43 fractures: orbitozygomatic (n=6); zygomatic (n=1); maxillary (n=6); Le Fort I (n=1); Le Fort II (n=1); mandibular (n=28).
**Rojas et al. 2002 [22]**	Santiago, Chile. Private trauma hospital (Hospital del Trabajador de Santiago) / Maxillofacial Surgery Unit / Retrospective observational hospital-based study (January 1990 - December 1996).	160 adult patients with mandibular fractures.	Mean 33 years (range 14-65).	Male 90.6%; Female 9.4%.	Traffic accidents 46.1%; falls 20.4%; interpersonal violence 13.1%; other causes 14.7%; unspecified 5.7%.	Distribution of 245 mandibular fractures: subcondylar 34.6%; angle 20.0%; body 17.1%; parasymphysis 16.3%; symphysis 7.8%; ramus 2.9%; coronoid process 0.8%; intracapsular condyle 0.4%.
**Secchi et al. 2021 [23]**	Santiago, Chile. Public tertiary hospital (Complejo Asistencial Barros Luco, CABL) / Dental Service, Emergency Unit / Retrospective observational study based on Emergency Care Records (DAU) (January 2018 - June 2019)	189 adult patients with midface maxillofacial trauma.	Mean 42 ± 21 years. Age groups: 12-19 (8.46%); 20-29 (28.57%); 30-39 (19.57%); 40-49 (13.70%); 50-59 (8.46%); 60-69 (5.82%); 70-79 (7.93%); 80-89 (4.76%); 90-99 (2.64%).	Male 70.4%; Female 29.6%.	Home accidents / falls 38.6%; interpersonal violence 29.1%; traffic accidents 6.3%; other causes 22.8%; school accidents 3.2%.	Distribution of 227 fractures: nasal (n=105); orbitozygomatic complex (n=28); orbital (n=26); maxilla (n=25); maxillomalar (n=20); zygomatic (n=18); NOE (n=3); Le Fort II (n=2).
**Soto et al. 2023 [24]**	Santiago, Chile. Instituto Traumatológico Dr. Teodoro Gebauer / Maxillofacial Surgery Unit / Retrospective	84 patients with surgical indication for maxillofacial trauma (pre-pandemic	Mean 38 ± 15.9 years (pre-pandemic 40 ±	Male 82.1%; Female 17.9% (male proportion	Interpersonal violence 52.4%; traffic accidents 13.1%; falls (height +	Pre-pandemic (n=64 fractures): mandibular (n=25); zygomatic (n=19); orbital (n=11); maxillary (n=4); nasal (n=2); panfacial (n=2); NOE

	observational comparative study (March 2019-February 2020 vs March 2020-February 2021).	n=53; pandemic n=31).	18.0; pandemic 36 ± 11.8).	increased from 79.3% to 87.1% during pandemic).	bicycle) 20.2%; sports-related trauma 4.8%; firearm injury 2.4%; other causes (objects, animals) 7.1%.	(n=1). Pandemic (n=41 fractures): mandibular (n=15); zygomatic (n=14); orbital (n=10); maxillary (n=2); nasal (n=0); panfacial (n=0); NOE (n=0).
**Tapia-Contreras et al. 2025 [25]**	Rancagua, Chile. Public tertiary hospital (Dr. Franco Ravera Zunino Hospital) / Maxillofacial Surgery Unit / Retrospective observational study based on surgical protocols (July 2017 - June 2022).	368 surgically treated maxillofacial fractures.	Mean 36 years (range 2-84).	Male 80.4%; Female 19.6%.	Not reported.	Mandibular (n=131); orbito-zygomatic-maxillary complex (n=112); panfacial (n=48); maxillary (n=28); zygomatic (n=17); orbital (n=18); NOE (n=11); frontal (n=3).
**Vázquez et al. 2021[26]**	Santiago, Chile. Public tertiary hospital (Complejo Asistencial Barros Luco) / Emergency Department / Retrospective observational study (January 2018 - June 2019).	57 patients with mandibular fractures attended at emergency service.	Mean 31 ± 12 years (range 16-67). Age groups: <20 (15.8%); 20-29 (38.6%); 30-39 (24.6%); 40-49 (14.0%); 50-59 (3.5%); ≥60 (3.5%).	Male 80.7%; Female 19.3%.	Domestic accidents 36.8%; interpersonal violence 35.1%; traffic accidents 26.3%; school accidents 1.8%.	Mandibular fractures only (n=82): angle (n=29); symphysis/parasymphysis (n=16); condyle (n=14); mandibular body (n=14); mandibular ramus (n=9).
**Venegas et al. 2013 [19]**	La Serena, Chile. Public regional hospital (Hospital San Juan de Dios de La Serena) / Emergency Department and Maxillofacial Surgery referral unit / Retrospective descriptive case series (July 2004 - July 2011).	194 patients with maxillofacial trauma.	Mean 27 years (range 0-87). Age groups: 0-10 (19.5%); 11-20 (25.1%); 21-30 (20.7%); 31-40 (14.0%); 41-50 (10.2%); 51-60 (4.4%); ≥61 (6.1%).	Male 75.2%; Female 24.8%.	Interpersonal violence 36%; falls 27%; sports-related trauma 16%; other causes 21%.	Distribution of 194 fractures (dentoalveolar excluded): orbital (n=20); zygomatic (n=42); nasal (n=15); maxillary (n=10); mandibular (n=107).
**Werlinger et al. 2019 [27]**	Central Chile (Santiago Metropolitan Region and Valparaíso Region). Public tertiary hospitals (Hospital Dr. Sótero del Río, Hospital Carlos Van Buren, Hospital Dr. Gustavo Fricke) / Adult Emergency Departments / Multicenter prospective observational study based on emergency	2,485 adult patients (≥18 years) with maxillofacial trauma.	Median 34 years (IQR 25-51). Age groups: 18-29 (37.1%); 30-39 (16.6%); 40-49 (13.5%); 50-59 (12.2%);	Male 63.5%; Female 36.5%.	Violence 42.3%; falls 13.1%; road traffic accidents 12.9%; other causes 31.7%; not reported / undetermined 33.3% of total cases.	3,096 injuries recorded; soft-tissue injuries (n=2,206). Fractures: nasal (n=474); malar/maxillary (n=156); mandibular (n=120); other skull/facial bones (n=93); orbital (n=38); skull base (n=5); unspecified facial/skull (n=4).

	registries (May 2016 - April 2017).		≥60 (20.6%).			
**Zapata et al. 2015 [28]**	Santiago, Chile. National referral trauma center (Instituto Traumatológico Dr. Teodoro Gebauer Weisser) / Maxillofacial Surgery Unit / Retrospective observational study (January 2001 - December 2010).	240 adult patients surgically treated for mandibular fractures.	Mean 30.3 years. Age groups: 10-19 (14.6%); 20-29 (43.3%); 30-39 (21.3%); 40-49 (12.9%); 50-59 (5.4%); ≥60 (2.5%).	Male 85.4%; Female 14.6%.	Interpersonal violence 72.1%; traffic accidents 11.3%; pedestrian accidents 4.6%; falls from height 4.2%; bicycle accidents 3.8%; sports-related trauma 2.9%; other causes 1.3%.	Mandibular fractures only (n=353): angle 42.8%; body 37.1%; condyle 14.7%; symphysis 2.3%; ramus 2.5%; coronoid 0.6%.

Supplementary Table S4. Risk of Bias assessment

Study	JBI critical appraisal tool for SRs addressing questions of prevalence								
	**D1**	**D2**	**D3**	**D4**	**D5**	**D6**	**D7**	**D8**	**D9**
Collao-Gonzalez et al. 2014	Unclear	Yes	Yes	Yes	Unclear	Yes	Unclear	No	
Cuellar et al. 2019	Unclear	Unclear	Unclear	Yes	Unclear	Yes	Unclear	No	
Duarte 1999	Unclear	Unclear	Unclear	Yes	Unclear	Unclear	Unclear	No	
Espinosa et al. 2019	Unclear	Yes	Unclear	Yes	No	Yes	Yes	No	
Faille & Badillo, 2018	Unclear	Unclear	Unclear	Yes	No	Unclear	Unclear	No	
De la Fuente et al. 2023	Unclear	Unclear	Unclear	Yes	Unclear	Unclear	Unclear	No	
Gonzalez et al. 2015	Unclear	Yes	Unclear	Yes	Unclear	Unclear	Unclear	No	
Medina et al. 2006	Unclear	Yes	Yes	Yes	Yes	Unclear	Unclear	No	
Quitral et al. 2022	Unclear	Unclear	Unclear	Yes	Unclear	Unclear	Unclear	No	
Raposo et al. 2013	Unclear	Yes	Unclear	Yes	No	Unclear	Unclear	No	NA
Raposo et al. 2021	Unclear	Yes	Unclear	Yes	No	Unclear	Unclear	No	
Rojas et al. 2002	Unclear	Yes	Unclear	Yes	Unclear	Unclear	Unclear	No	
Secchi et al. 2021	Unclear	Yes	Unclear	Yes	No	Unclear	No	No	
Soto et al. 2023	Unclear	Yes	Unclear	Yes	No	Yes	Yes	No	
Tapia-Contreras et al. 2025	Unclear	Yes	Unclear	Yes	Unclear	Unclear	Unclear	No	
Vázquez et al. 2021	Unclear	Yes	Unclear	Yes	No	Unclear	Unclear	No	
Venegas et al. 2013	Unclear	Yes	Unclear	Yes	Unclear	Yes	Unclear	No	
Werlinger et al. 2019	Unclear	Yes	Yes	Yes	Unclear	Yes	Yes	No	
Zapata et al. 2015	Unclear	Yes	Unclear	Yes	No	Yes	Unclear	No	

Color coding: Yes No Unclear NA: Not applicable

Appraisal tool dimensions

D1: Was the sample frame appropriate to address the target population?

D2: Were study participants sampled in an appropriate way?

D3: Was the sample size adequate?

D4: Were the study subjects and the setting described in detail?

D5: Was the data analysis conducted with sufficient coverage of the identified sample?

D6: Were valid methods used for the identification of the condition?

D7: Was the condition measured in a standard, reliable way for all participants?

D8: Was there appropriate statistical analysis?

D9: Was the response rate adequate, and if not, was the low response rate managed appropriately?

Supplementary Table S5. Summary of Findings table

Outcome	N° of studies (total sample size)	Study design	Risk of Bias	Indirectness	Inconsistency	Imprecision	Publication bias	Overall estimate [95% confidence interval]	Certainty of the evidence
**Proportion of fractures:**									
**Frontal**	5 (1218)	Registry-based observational cohort studies	↓	—	↓	—	—	2.56% [0.00 to 9.52]	⊕⊕◯◯ **Low**
**Orbital**	11 (3037)		↓	—	↓	—	—	7.63% [3.30 to 13.47]	⊕⊕◯◯ **Low**
**Zygomatic**	12 (2377)		↓	—	↓	—	—	24.23% [18.58 to 30.35]	⊕⊕◯◯ **Low**
**Nasal**	6 (1794)		↓	—	↓↓	↓	—	16.25% [0.11 to 49.11]	⊕◯◯◯ **Very low**
**Maxillary**	11 (2915)		↓	—	↓	—	—	8.60% [5.16 to 12.78]	⊕⊕◯◯ **Low**
**Mandibular**	12 (2880)		↓	—	↓↓	↓	—	45.27% [31.02 to 59.92]	⊕◯◯◯ **Very low**
**Le Fort**	7 (1683)		↓	—	↓	—	—	4.31% [1.55 to 8.22]	⊕⊕◯◯ **Low**
**NOE**	7 (1679)		↓	—	↓	↓	—	6.07% [0.00 to 23.95]	⊕◯◯◯ **Very low**
**Panfacial**	5 (1266)		↓	—	↓	—	—	4.24% [0.48 to 10.97]	⊕⊕◯◯ **Low**

Figures

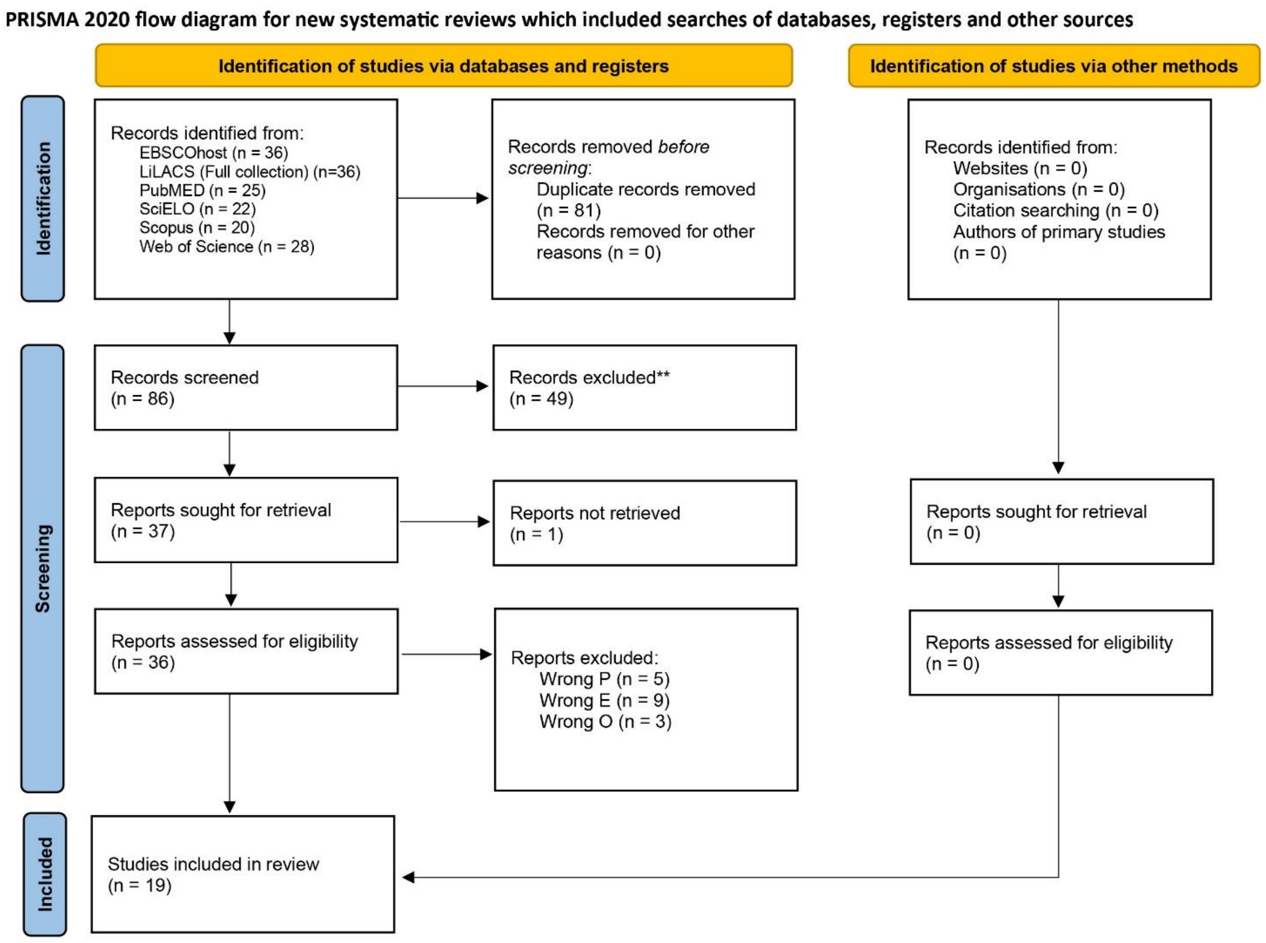

Supplementary Figure S1. PRISMA 2020 flow diagram for the systematic searching process.

Study or Subgroup	Events	Total	Events per 100 observations IV, Random, 95% CI
**Frontal**			
Cuellar et al. 2019	1	255	0.39 [0.01; 2.17]
Gonzalez et al. 2015	40	403	9.93 [7.19; 13.27]
Medina et al. 2006	2	27	7.41 [0.91; 24.29]
Raposo et al. 2013	2	165	1.21 [0.15; 4.31]
Tapia et al. 2025	3	368	0.82 [0.17; 2.36]
**Total (95% CI)**		**1218**	**2.56 [0.00; 9.52]**
Heterogeneity: Tau^2 = 0.0120; Chi^2 = 57.57, df = 4 (P < 0.0001); I^2 = 93.1%			
**Mandibular**			
Cuellar et al. 2019	200	255	78.43 [72.87; 83.32]
Faille & Badillo 2018	210	414	50.72 [45.80; 55.64]
De la Fuente et al. 2023	105	176	59.66 [52.01; 66.97]
Gonzalez et al. 2015	60	403	14.89 [11.56; 18.74]
Medina et al. 2006	6	27	22.22 [8.62; 42.26]
Raposo et al. 2013	121	165	73.33 [65.90; 79.91]
Raposo et al. 2021	28	43	65.12 [49.07; 78.99]
Soto et al. 2023 (pre−pandemic)	25	64	39.06 [27.10; 52.07]
Soto et al. 2023 (pandemic)	15	41	36.59 [22.12; 53.06]
Tapia et al. 2025	131	368	35.60 [30.70; 40.73]
Venegas et al. 2013	107	194	55.15 [47.87; 62.28]
Werlinger et al. 2019	120	730	16.44 [13.82; 19.33]
**Total (95% CI)**		**2880**	**45.27 [31.02; 59.92]**
Heterogeneity: Tau^2 = 0.0507; Chi^2 = 661.71, df = 11 (P < 0.0001); I^2 = 98.3%			
**Zygomatic**			
Cuellar et al. 2019	31	255	12.16 [8.41; 16.81]
Faille & Badillo 2018	137	414	33.09 [28.57; 37.85]
De la Fuente et al. 2023	49	176	27.84 [21.36; 35.08]
Gonzalez et al. 2015	136	403	33.75 [29.14; 38.59]
Medina et al. 2006	2	27	7.41 [0.91; 24.29]
Raposo et al. 2013	31	165	18.79 [13.14; 25.60]
Raposo et al. 2021	7	43	16.28 [6.81; 30.70]
Secchi et al. 2021	46	227	20.26 [15.23; 26.09]
Soto et al. 2023 (pre−pandemic)	19	64	29.69 [18.91; 42.42]
Soto et al. 2023 (pandemic)	14	41	34.15 [20.08; 50.59]
Tapia et al. 2025	129	368	35.05 [30.18; 40.17]
Venegas et al. 2013	42	194	21.65 [16.07; 28.12]
**Total (95% CI)**		**2377**	**24.23 [18.58; 30.35]**
Heterogeneity: Tau^2 = 0.0088; Chi^2 = 88.61, df = 11 (P < 0.0001); I^2 = 87.6%			
**Orbital**			
Cuellar et al. 2019	4	255	1.57 [0.43; 3.97]
Faille & Badillo 2018	4	414	0.97 [0.26; 2.46]
De la Fuente et al. 2023	7	176	3.98 [1.61; 8.02]
Gonzalez et al. 2015	88	403	21.84 [17.90; 26.19]
Raposo et al. 2013	5	165	3.03 [0.99; 6.93]
Secchi et al. 2021	26	227	11.45 [7.62; 16.33]
Soto et al. 2023 (pre−pandemic)	11	64	17.19 [8.90; 28.68]
Soto et al. 2023 (pandemic)	10	41	24.39 [12.36; 40.30]
Tapia et al. 2025	18	368	4.89 [2.92; 7.62]
Venegas et al. 2013	20	194	10.31 [6.41; 15.47]
Werlinger et al. 2019	38	730	5.21 [3.71; 7.08]
**Total (95% CI)**		**3037**	**7.63 [3.30; 13.47]**
Heterogeneity: Tau^2 = 0.0176; Chi^2 = 180.19, df = 10 (P < 0.0001); I^2 = 94.5%			
**Nasal**			
Faille & Badillo 2018	22	414	5.31 [3.36; 7.94]
Raposo et al. 2013	1	165	0.61 [0.02; 3.33]
Secchi et al. 2021	105	227	46.26 [39.64; 52.97]
Soto et al. 2023 (pre−pandemic)	2	64	3.12 [0.38; 10.84]
Venegas et al. 2013	15	194	7.73 [4.39; 12.43]
Werlinger et al. 2019	474	730	64.93 [61.35; 68.40]
**Total (95% CI)**		**1794**	**16.25 [0.11; 49.11]**
Heterogeneity: Tau^2 = 0.1161; Chi^2 = 856.76, df = 5 (P < 0.0001); I^2 = 99.4%			
**Maxillary**			
Cuellar et al. 2019	13	255	5.10 [2.74; 8.56]
Faille & Badillo 2018	29	414	7.00 [4.74; 9.91]
De la Fuente et al. 2023	7	176	3.98 [1.61; 8.02]
Gonzalez et al. 2015	25	403	6.20 [4.05; 9.02]
Raposo et al. 2021	6	43	13.95 [5.30; 27.93]
Secchi et al. 2021	45	227	19.82 [14.84; 25.61]
Soto et al. 2023 (pre−pandemic)	4	64	6.25 [1.73; 15.24]
Soto et al. 2023 (pandemic)	2	41	4.88 [0.60; 16.53]
Tapia et al. 2025	28	368	7.61 [5.12; 10.81]
Venegas et al. 2013	10	194	5.15 [2.50; 9.28]
Werlinger et al. 2019	156	730	21.37 [18.45; 24.52]
**Total (95% CI)**		**2915**	**8.60 [5.16; 12.78]**
Heterogeneity: Tau^2 = 0.0087; Chi^2 = 132.19, df = 10 (P < 0.0001); I^2 = 92.4%			
**NOE**			
Faille & Badillo 2018	21	414	5.07 [3.17; 7.65]
De la Fuente et al. 2023	1	176	0.57 [0.01; 3.12]
Gonzalez et al. 2015	13	403	3.23 [1.73; 5.45]
Medina et al. 2006	17	27	62.96 [42.37; 80.60]
Secchi et al. 2021	3	227	1.32 [0.27; 3.81]
Soto et al. 2023 (pre−pandemic)	1	64	1.56 [0.04; 8.40]
Tapia et al. 2025	11	368	2.99 [1.50; 5.29]
**Total (95% CI)**		**1679**	**6.07 [0.00; 23.95]**
Heterogeneity: Tau^2 = 0.0692; Chi^2 = 71.45, df = 6 (P < 0.0001); I^2 = 91.6%			
**Le Fort**			
Cuellar et al. 2019	6	255	2.35 [0.87; 5.05]
Faille & Badillo 2018	50	414	12.08 [9.10; 15.61]
De la Fuente et al. 2023	6	176	3.41 [1.26; 7.27]
Gonzalez et al. 2015	29	403	7.20 [4.87; 10.17]
Raposo et al. 2013	5	165	3.03 [0.99; 6.93]
Raposo et al. 2021	2	43	4.65 [0.57; 15.81]
Secchi et al. 2021	2	227	0.88 [0.11; 3.15]
**Total (95% CI)**		**1683**	**4.31 [1.55; 8.22]**
Heterogeneity: Tau^2 = 0.0066; Chi^2 = 51.17, df = 6 (P < 0.0001); I^2 = 88.3%			
**Panfacial**			
Cuellar et al. 2019	2	255	0.78 [0.10; 2.80]
De la Fuente et al. 2023	8	176	4.55 [1.98; 8.76]
Gonzalez et al. 2015	12	403	2.98 [1.55; 5.14]
Soto et al. 2023 (pre−pandemic)	2	64	3.12 [0.38; 10.84]
Tapia et al. 2025	48	368	13.04 [9.78; 16.92]
**Total (95% CI)**		**1266**	**4.24 [0.48; 10.97]**
Heterogeneity: Tau^2 = 0.0097; Chi^2 = 51.75, df = 4 (P < 0.0001); I^2 = 92.3%			

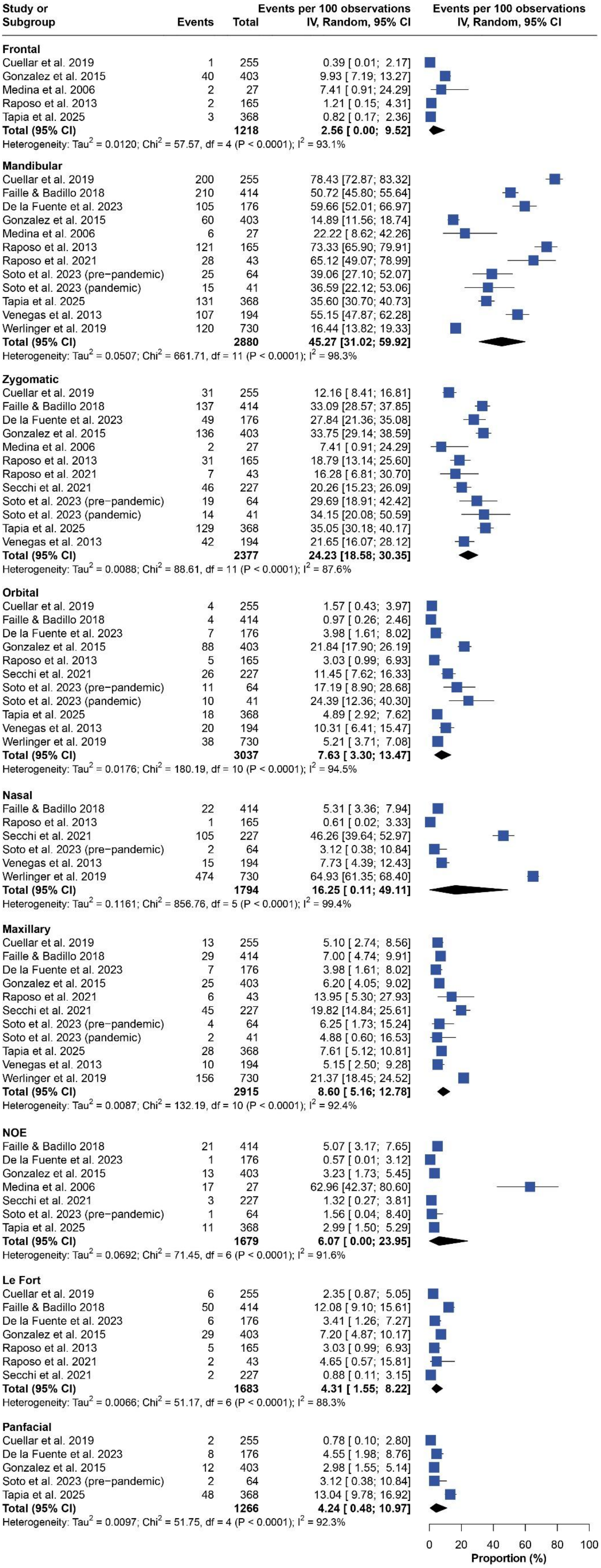

Suplementary Figure S2. Forest plot of single-proportion random-effects meta-analysis of the prevalence of maxillofacial trauma in Chile. CI: Confidence Interval, IV: Inverse Variance.

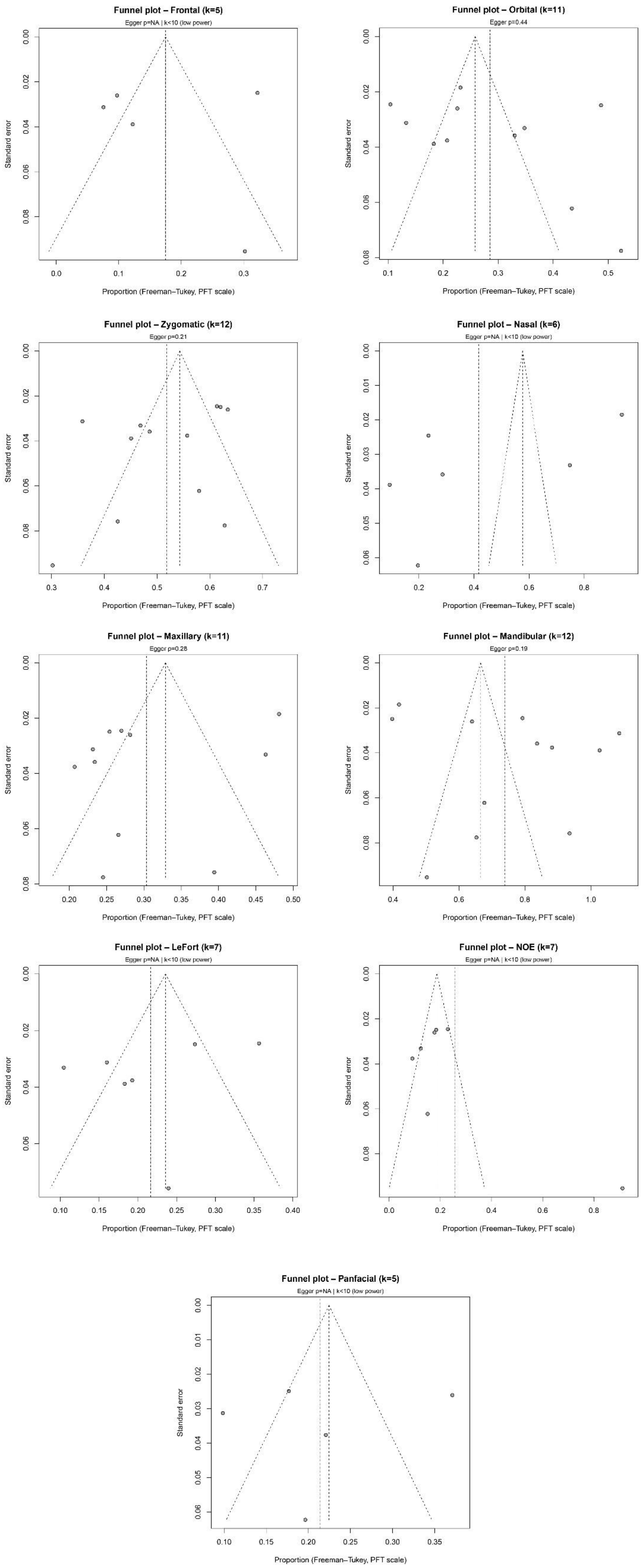

Suplementary Figure S3. Funnel plot of single-proportion random-effects meta-analysis of the prevalence maxillofacial trauma in Chile.
