# Supplementary material for: Epidemiology of Craniomaxillofacial Trauma in Chile: A Systematic Review and 24-Year Nationwide Interrupted Time-Series Analysis
Source: Craniomaxillofac Trauma Reconstr. 2026 Jul 3;19(3):32. doi: 10.3390/cmtr19030032 (PMC13398010; doi:10.3390/cmtr19030032)
Supplement: Supplementary file 1 [file cmtr-19-00032-s001.zip › Supplementary-File S2.pdf]

Supplementary File 2

Methods for the Nationwide administrative database study (DEIS)

Introduction

The DEIS database includes mandatory records of all hospital discharges from public and private healthcare institutions in Chile and contains anonymized information on patient demographics, diagnoses coded according to the International Classification of Diseases, 10th Revision (ICD-10), length of hospital stay, discharge status, and external causes of injury when available. Craniomaxillofacial trauma (CMFt) cases were defined as discharges with ICD-10 codes indicating facial soft tissue injuries or facial bone fractures. For the present study, all hospital discharges between January 2001 and December 2024 with a primary diagnosis corresponding to traumatic injuries (ICD-10 codes S00–S99 and T00–T14) were identified. Dentoalveolar trauma and isolated intraoral injuries were excluded to ensure consistency with the systematic review definitions.

Operational assumptions and data handling. In the DEIS open discharge files, diagnosis codes are available at the four-character ICD-10 level (e.g., S022, corresponding to S02.2), and may appear with minor formatting differences across years (e.g., punctuation or extra whitespace). Accordingly, diagnosis fields were standardized by trimming whitespace, converting to uppercase, and removing dots when present, while preserving the original four-character coding available in the source files. The CMFt definition was applied to the primary diagnosis (DIAG1) to align with the principal discharge diagnosis in administrative data. Discharge records were analyzed as episodes of inpatient care; repeated admissions for the same patient could not be linked or de-duplicated due to anonymization. Core variables used in this workflow included year of discharge, sex, an age-group field harmonized into categorical age bands, region and comuna of residence, length of stay, and discharge status. External causes of injury (DIAG2) were used when available to classify etiology; however, missingness and heterogeneous completeness of external-cause coding across years and institutions were expected, and etiology-specific analyses therefore distinguished between known versus missing/unspecified external causes.

Table S1. ICD-10 Codes related to CMFt

CMFt domain	ICD-10 code	Clinical label	Notes
Soft tissue injuries			
	S001	Contusion of eyelid and periocular area	
	S003	Superficial injury of nose	
	S004	Superficial injury of ear	
	S005	Superficial injury of lip and oral cavity	
	S012	Open wound of nose	
	S013	Open wound of ear	
	S014	Open wound of cheek and temporomandibular area	
	S015	Open wound of lip and oral cavity	
Facial bone fractures (hard tissue)			

CMFt domain	ICD-10 code	Clinical label	Notes
	S020	Fracture of vault of skull	Frontal and parietal bone
	S022	Fracture of nasal bones	
	S023	Fracture of orbital floor	
	S024	Fracture of malar and maxillary bones	Maxillary, zygomatic and zygomatic process of temporal bone
	S026	Fracture of mandible	
	S027	Multiple fractures involving skull and facial bones	
	S028	Other specified fractures of skull and facial bones	Medial and lateral orbital wall, right or left side
	S029	Fracture of skull and facial bones, part unspecified	

0. Packages and configuration

All analyses were conducted in R. The following packages were used:

Data access and database back-end

- `DBI`, `duckdb`: interface and embedded database engine used to query yearly DEIS CSV files efficiently and reproducibly.
- `arrow`: optional parquet for saving intermediate datasets in a columnar format.

Data manipulation and reporting utilities

- `dplyr`, `tibble`, `tidyr`, `stringr`: core tidyverse tools for data cleaning, transformation, reshaping, and text normalization (e.g., ICD-10 and region codes).

- `cli`: lightweight progress bars and console messages to support quality assurance during long yearly ingestion loops.

Statistical modeling and inference

- `MASS`: negative binomial regression (glm.nb) for count-based interrupted time series (ITS) models when overdispersion is present.
- `broom`: consistent extraction of fitted values and uncertainty (e.g., model augmentation for prediction bands).
- `lmtest`, `sandwich`: robust (heteroskedasticity-consistent) standard errors for linear-model inference (coeftest with HC-type variance estimators).
- `mvtnorm`: used for multivariate normal utilities when needed (e.g., simulation-based steps), included here for completeness.

Visualization

- `ggplot2`: all figures and diagnostic plots.
- `scales`: axis formatting (percentages, etc.) and transformations.

Geospatial mapping

- `chilemapas`: region shapefiles and geographic helpers for Chile.
- `sf`: simple features framework used by ggplot2::geom_sf().

To ensure portability across machines, package installation/loading was standardized using `pacman`:

```r
if (!require("pacman")) install.packages("pacman")
pacman::p_load(
  DBI, duckdb, dplyr, arrow, cli,
  lmtest, sandwich, MASS, broom,
  ggplot2, mvtnorm, tibble, tidyr,
  scales, stringr
)
```

Configuration

This section defines the project inputs, output paths, study period, and the ICD-10 code sets used to operationalize CMFt. It also pre-specifies the intervention periods required for interrupted time series (ITS) analyses. This structure implements a segmented regression with: * an underlying pre-period secular trend, * a COVID-period indicator effect, and * a post-2022 indicator plus post-2022 slope term.

```r
# Directory
RAW_DIR <- "C:/Users/gusta/Downloads/Estudio tendencias fx maxilo/data_raw"
PROJECT_DIR <- "C:/Users/gusta/Downloads/Estudio tendencias fx maxilo"
OUT_DIR_PARQUET <- file.path(PROJECT_DIR, "data_processed", "base_parquet")
dir.create(OUT_DIR_PARQUET, recursive = TRUE, showWarnings = FALSE)

# Time period
YEARS <- 2001:2024
```

```r
# CMFt domain codes
CMF_SOFT_SET <- c("S001","S003","S004","S005","S012","S013","S014","S015")
CMF_HARD_SET <- c("S020", "S022","S023","S024","S026","S027","S028","S029")

file_for_year <- function(y) file.path(RAW_DIR, paste0("EGRE_DATOS_ABIERTOS_", y, ".csv"))

# Period definitions (ITS)
COVID_YEARS <- c(2020L, 2021L)
POST_YEAR   <- 2022L
```

1. Utility functions

This subsection defines helper functions used consistently across analyses to (i) compute robust inference, (ii) stabilize proportion outcomes, and (iii) generate ITS regressors, predictions, and standardized plots.

Robust inference for linear models

This produces coefficient tests using heteroskedasticity-consistent (HC) variance estimators (default HC1). It is used when fitting linear models (e.g., mean LOS or logit-transformed proportions) to provide robust standard errors, which are less sensitive to non-constant variance over time.

```r
coeftest_robust <- function(model, type = "HC1") {
  lmtest::coeftest(model, vcov = sandwich::vcovHC(model, type = type))
}
```

Stabilizing proportion outcomes

Some outcomes are proportions (e.g., annual proportion with LOS 5). When applying a logit transform, values of exactly 0 or 1 produce infinite logits. `clamp01()` bounds proportions to `[eps, 1-eps]` before `qlogis()`. This is a pragmatic numerical stabilization step; in interpretation, it should be understood as avoiding undefined values rather than altering the underlying counts.

```r
clamp01 <- function(p, eps = 0.001) pmin(pmax(p, eps), 1 - eps)
```

ITS regressor construction

For each calendar year y, the function creates:

- **Centered time index (pre-intervention secular trend)**

$$t_y = y - y_{\min}$$

- **Pandemic indicator (additive level difference during COVID years)**

$$\text{covid}_y = \begin{cases} 1, & y \in \{2020, 2021\} \\ 0, & \text{otherwise} \end{cases}$$

- **Post-acute indicator (level change starting in 2022)**

$$\text{post}_y = \begin{cases} 1, & y \geq 2022 \\ 0, & y < 2022 \end{cases}$$

- **Post-period slope term (trend change after 2022)**

$$t_{\text{post},y} = \begin{cases} y - 2022, & y \geq 2022 \\ 0, & y < 2022 \end{cases}$$

This corresponds to segmented regression of the form:

$$g\big(\mathbb{E}[Y_y]\big) = \beta_0 + \beta_1 t_y + \beta_2 \text{covid}_y + \beta_3 \text{post}_y + \beta_4 t_{\text{post},y}.$$

where $g(\cdot)$ is either the **identity link** (linear outcomes) or the **logit link** (proportion outcomes), as implemented below.

```
make_its_vars <- function(df, year_col = "ANO_EGRESO", post_year = POST_YEAR, covid_years = COVID_YEARS)
  y <- df[[year_col]]
  df %>%
    mutate(
      t        = y - min(y, na.rm = TRUE),
      covid    = if_else(y %in% covid_years, 1L, 0L),
      post     = if_else(y >= post_year, 1L, 0L),
      t_post   = if_else(y >= post_year, y - post_year, 0L)
    )
}
```

Generic ITS linear model with optional logit transformation

- If `transform = "none"`: Y_y is modeled on its original scale using an ordinary least squares (OLS) segmented regression. Predictions and 95% confidence intervals (CIs) are computed as:

$$\hat{Y}_y \pm 1.96 \cdot \text{SE}(\hat{Y}_y).$$

- If `transform = "logit"`: the annual proportion p_y is mapped to log-odds via the logit transform,

$$\text{logit}(p_y) = \log\left(\frac{p_y}{1 - p_y}\right),$$

fitted using OLS on the linear predictor scale, and then back-transformed using the inverse logit to yield fitted proportions $\hat{p}_y$:

$$\text{logit}^{-1}(\eta_y) = \frac{1}{1 + e^{-\eta_y}}, \qquad \hat{p}_y = \text{logit}^{-1}(\hat{\eta}_y).$$

The 95% confidence bands are computed on the linear predictor scale and then inverse-logit transformed:

$$\hat{\eta}_y \pm 1.96 \cdot \text{SE}(\hat{\eta}_y) \quad \Longrightarrow \quad \text{logit}^{-1}\big(\hat{\eta}_y \pm 1.96 \cdot \text{SE}(\hat{\eta}_y)\big).$$

```r
fit_its_lm <- function(df, y, transform = c("none", "logit"),
                       year_col = "ANO_EGRESO", post_year = POST_YEAR, covid_years = COVID_YEARS) {
  transform <- match.arg(transform)

  dat <- df %>%
    make_its_vars(year_col = year_col, post_year = post_year, covid_years = covid_years) %>%
    mutate(.y_raw = .data[[y]])

  if (transform == "logit") {
    dat <- dat %>% mutate(.y = qlogis(clamp01(.y_raw)))
    model <- lm(.y ~ t + covid + post + t_post, data = dat)
    pred  <- broom::augment(model, newdata = dat, se_fit = TRUE) %>%
      transmute(
        !!year_col := .data[[year_col]],
        y_obs = .y_raw,
        y_fit = plogis(.fitted),
        y_lo  = plogis(.fitted - 1.96 * .se.fit),
        y_hi  = plogis(.fitted + 1.96 * .se.fit)
      )
  } else {
    dat <- dat %>% mutate(.y = .y_raw)
    model <- lm(.y ~ t + covid + post + t_post, data = dat)
    pred  <- broom::augment(model, newdata = dat, se_fit = TRUE) %>%
      transmute(
        !!year_col := .data[[year_col]],
        y_obs = .y_raw,
        y_fit = .fitted,
        y_lo  = .fitted - 1.96 * .se.fit,
        y_hi  = .fitted + 1.96 * .se.fit
      )
  }

  list(model = model, pred = pred, transform = transform, outcome = y)
}
```

Standardized ITS plotting

Plot elements

- **Points**: observed annual values (y_obs)
- **Line**: fitted ITS predictions (y_fit)
- **Ribbon**: 95% confidence interval (y_lo, y_hi)
- **Vertical dotted lines**: key boundaries (default: 2019 and 2022) for visual reference

```r
plot_its <- function(pred_df, year_col = "ANO_EGRESO",
                     ylab = "Outcome", title = "Interrupted time series",
                     subtitle = NULL, percent = FALSE,
                     vlines = c(2019, 2022)) {
  p <- ggplot(pred_df, aes(x = .data[[year_col]])) +
    geom_ribbon(aes(ymin = y_lo, ymax = y_hi), alpha = 0.25) +
    geom_line(aes(y = y_fit), linewidth = 1.05) +
    geom_point(aes(y = y_obs), size = 2, alpha = 0.85) +
```

```r
    geom_vline(xintercept = vlines, linetype = "dotted") +
    labs(x = "Year", y = ylab, title = title, subtitle = subtitle) +
    theme_minimal(base_size = 13)

  if (percent) {
    p <- p + scale_y_continuous(labels = scales::percent_format(accuracy = 1))
  }
  p
}
```

Etiology mapping

This section operationalizes injury etiology using the external cause of injury field (`DIAG2`) when available. In DEIS open discharge data, `DIAG2` may be missing, inconsistently formatted, or recorded with punctuation/whitespace. We therefore: (i) normalize the code string, (ii) classify each record into a granular ICD-10 external-cause group (`ext_cause_grp`), and (iii) collapse these granular groups into a 6-category etiology taxonomy (`ext_cause_6grp`) for trend and burden analyses.

Code normalization and parsing `DIAG2` is standardized to a compact format suitable for rule-based mapping:

- Converted to character and upper case (`toupper()`).
- Dots removed (`gsub("\\.", "", ...)`) and whitespace removed (`gsub("\\s+", "", ...)`).
- Only the first three characters are used to classify the external cause at the ICD-10 block level (e.g., `V01`, `W19`, `X85`).
- The first character (`diag2_L`) captures the ICD-10 chapter letter (`V`, `W`, `X`, `Y`), and the following two digits (`diag2_N`) capture the numeric range used to assign groups.

Assumption: Etiology coding is treated as valid if the first three characters correspond to a recognizable ICD-10 external cause block. Records with missing/blank `DIAG2` are treated as missing etiology and are not forced into a specific mechanism.

Granular external-cause grouping: `ext_cause_grp` Each record is mapped to one mutually exclusive category using ICD-10 external cause blocks:

- Transport injuries: `V01-V99`
- Falls: `W00-W19`
- Mechanical forces (inanimate): `W20-W49`
- Mechanical forces (animate): `W50-W64`
- Drowning/submersion: `W65-W74`
- Electric current/radiation/extreme temperatures: `W85-W99`
- Fire/heat/burns: `X00-X19`
- Contact with venomous animals/plants: `X20-X29`
- Forces of nature: `X30-X39`
- Accidental poisonings: `X40-X49`
- Overexertion/strenuous movement: `X50-X57`
- Unspecified/other specified accidents: `X58-X59`
- Intentional self-harm: `X60-X84`
- Assault: `X85-Y09`
- Undetermined intent: `Y10-Y34`
- Adverse events/complications of medical and surgical care: `Y40-Y84`

- Sequelae of external causes: `Y85-Y89`
- Supplementary factors: `Y90-Y99`

Remaining codes are flagged as **Other/Unclassifiable** for review.

Statistical handling of missingness: If `DIAG2` is missing or blank, `ext_cause_grp` is set to `NA` (missing). This preserves the fact that external-cause coding is not complete in administrative discharge data and prevents implicit assumptions about mechanism.

Collapsing to 6 etiology categories: `ext_cause_6grp` For interpretability and stable time-series estimation, `ext_cause_grp` is collapsed into six broader categories:

1. **Low-energy accidental injuries**
   Falls (`W00-W19`), mechanical forces inanimate (`W20-W49`), mechanical forces animate (`W50-W64`), and overexertion (`X50-X57`).
2. **Transport-related accidental injuries (high-energy)**
   Transport injuries (`V01-V99`).
3. **Interpersonal violence**
   Assault (`X85-Y09`).
4. **Unspecified mechanism of accidental injury**
   Unspecified/other specified accidents (`X58-X59`).
5. **Non-accidental injuries or complex external causes**
   Self-harm (`X60-X84`), undetermined intent (`Y10-Y34`), adverse events/complications of care (`Y40-Y84`), and sequelae (`Y85-Y89`).
   Rationale: these represent mechanisms not directly comparable to accidental injury energy categories and are often analytically grouped due to low counts or conceptual distinctness.
6. **Other rare external causes**
   Burns (`X00-X19`), electric current/radiation/extreme temperatures (`W85-W99`), forces of nature (`X30-X39`), venomous animals/plants (`X20-X29`), drowning (`W65-W74`), and accidental poisonings (`X40-X49`).

Finally, records that do not map cleanly (including missing `DIAG2`) are assigned to **No especificada/otras** as a dedicated level to enable explicit reporting of etiology missingness in descriptive tables and figures, rather than silently excluding those records.

```r
 Etiology mapping (DIAG2) -> ext_cause_grp + ext_cause_6grp
add_etiology_groups <- function(df) {
  df %>%
    mutate(
      DIAG2_clean = DIAG2 %>%
        as.character() %>%
        toupper() %>%
        gsub("\\.", "", .) %>%
        gsub("\\s+", "", .),
      diag2_3 = substr(DIAG2_clean, 1, 3),
      diag2_L = substr(diag2_3, 1, 1),
      diag2_N = suppressWarnings(as.integer(substr(diag2_3, 2, 3))),

      ext_cause_grp = case_when(
        is.na(DIAG2_clean) | DIAG2_clean == "" ~ NA_character_,
```

```
      diag2_L == "V" & diag2_N >= 1  & diag2_N <= 99 ~ "Transporte (V01-V99)",

      diag2_L == "W" & diag2_N >= 0  & diag2_N <= 19 ~ "Caídas (W00-W19)",

      diag2_L == "W" & diag2_N >= 20 & diag2_N <= 49 ~ "Fuerzas mecánicas inanimadas (W20-W49)",
      diag2_L == "W" & diag2_N >= 50 & diag2_N <= 64 ~ "Fuerzas mecánicas animadas (W50-W64)",

      diag2_L == "W" & diag2_N >= 65 & diag2_N <= 74 ~ "Ahogamiento/sumersión (W65-W74)",

      diag2_L == "W" & diag2_N >= 85 & diag2_N <= 99 ~ "Electricidad/radiación/temperaturas extremas

      diag2_L == "X" & diag2_N >= 0  & diag2_N <= 19 ~ "Fuego/calor/quemaduras (X00-X19)",

      diag2_L == "X" & diag2_N >= 20 & diag2_N <= 29 ~ "Animal/planta venenosa (X20-X29)",

      diag2_L == "X" & diag2_N >= 30 & diag2_N <= 39 ~ "Fuerzas de la naturaleza (X30-X39)",

      diag2_L == "X" & diag2_N >= 40 & diag2_N <= 49 ~ "Envenenamientos accidentales (X40-X49)",

      diag2_L == "X" & diag2_N %in% c(58, 59) ~ "Accidente inespecífico/otros especificados (X58-X59)

      diag2_L == "X" & diag2_N >= 50 & diag2_N <= 57 ~ "Sobreesfuerzo/esfuerzo físico (X50-X57)",

      diag2_L == "X" & diag2_N >= 60 & diag2_N <= 84 ~ "Autolesión (X60-X84)",

      (diag2_L == "X" & diag2_N >= 85 & diag2_N <= 99) |
        (diag2_L == "Y" & diag2_N >= 0  & diag2_N <= 9)  ~ "Agresiones (X85-Y09)",

      diag2_L == "Y" & diag2_N >= 10 & diag2_N <= 34 ~ "Intención no determinada (Y10-Y34)",

      diag2_L == "Y" & diag2_N >= 40 & diag2_N <= 84 ~ "Eventos adversos/complicaciones de atención (

      diag2_L == "Y" & diag2_N >= 85 & diag2_N <= 89 ~ "Secuelas de causas externas (Y85-Y89)",

      diag2_L == "Y" & diag2_N >= 90 & diag2_N <= 99 ~ "Factores suplementarios (Y90-Y99)",

      TRUE ~ "Otras/No clasificables (revisar DIAG2)"
    )
  ) %>%
  mutate(
    ext_cause_6grp = case_when(
      ext_cause_grp %in% c(
        "Caídas (W00-W19)",
        "Fuerzas mecánicas inanimadas (W20-W49)",
        "Fuerzas mecánicas animadas (W50-W64)",
        "Sobreesfuerzo/esfuerzo físico (X50-X57)"
      ) ~ "Low-energy accidental injuries",

      ext_cause_grp == "Transporte (V01-V99)" ~ "Transport-related accidental injuries (high-energy)"

      ext_cause_grp == "Agresiones (X85-Y09)" ~ "Interpersonal violence",

      ext_cause_grp == "Accidente inespecífico/otros especificados (X58-X59)" ~ "Unspecified mechanis
```

```
        ext_cause_grp %in% c(
          "Autolesión (X60-X84)",
          "Intención no determinada (Y10-Y34)",
          "Eventos adversos/complicaciones de atención (Y40-Y84)",
          "Secuelas de causas externas (Y85-Y89)"
        ) ~ "Non-accidental injuries or complex external causes",

        ext_cause_grp %in% c(
          "Fuego/calor/quemaduras (X00-X19)",
          "Electricidad/radiación/temperaturas extremas (W85-W99)",
          "Fuerzas de la naturaleza (X30-X39)",
          "Animal/planta venenosa (X20-X29)",
          "Ahogamiento/sumersión (W65-W74)",
          "Envenenamientos accidentales (X40-X49)"
        ) ~ "Other rare external causes",

        TRUE ~ NA_character_
      ),
      ext_cause_6grp = if_else(is.na(ext_cause_6grp) | ext_cause_6grp == "", "No especificada/otras", e
      ext_cause_6grp = factor(ext_cause_6grp)
    )
}
```

3. Database construction

To handle 24 yearly DEIS discharge files efficiently and reproducibly, we used **DuckDB** as an embedded analytical database. DuckDB allows SQL-based parsing and recoding directly from delimited text files without loading entire raw CSVs into memory, while still returning standard R data frames through **DBI**.

```
con <- dbConnect(
  duckdb::duckdb(),
  dbdir = file.path(PROJECT_DIR, "deis.duckdb"),
  read_only = FALSE
)
on.exit(dbDisconnect(con, shutdown = TRUE), add = TRUE)
```

Implementation details

- A persistent database file (`deis.duckdb`) is created under `PROJECT_DIR`. This improves workflow stability across sessions and supports iterative development.
- `read_only = FALSE` is used because DuckDB may create internal temporary objects during execution.
- `on.exit(..., add = TRUE)` guarantees that the connection is closed and the database is properly shutdown even if the script terminates early.

SQL extract

This section defines a year-specific SQL extractor that: (i) reads a single DEIS CSV file, (ii) standardizes key fields, (iii) restricts the cohort to trauma discharges based on primary diagnosis, and (iv) generates CMFt indicators (`cmf_group`, `is_cmf`) from predefined ICD-10 sets.

Reading and standardizing yearly CSV files The extractor uses `duckdb::read_csv_auto()` with conservative settings to maximize robustness across years (format changes, encoding differences, long lines, minor inconsistencies):

- `delim=';'` to match DEIS CSV formatting.
- `all_varchar=true` to ingest all columns as strings first, avoiding type inference errors.
- `strict_mode=false` and `ignore_errors=true` to tolerate malformed rows.
- `sample_size=-1` to improve inference reliability when auto-detecting columns (while still keeping them as varchar for safety).
- `max_line_size=10000000` to accommodate unusually long lines.

A key design assumption is that the DEIS "open" discharge files include at least the following fields (names may vary by year but are standardized in the SQL layer):

- `ANO_EGRESO`
- `SEXO`
- `GRUPO_EDAD`
- `COMUNA_RESIDENCIA`
- `REGION_RESIDENCIA`
- `DIAG1`
- `DIAG2`
- `DIAS_ESTADA`
- `CONDICION_EGRESO`

Defining the trauma base cohort (primary diagnosis only) Within each year, we define the "trauma base" cohort as discharges whose primary diagnosis (`DIAG1`) falls in:

- `S00-S99` (injuries to specific body regions), or
- `T00-T14` (injuries involving multiple and unspecified body regions).

Operationally, this is implemented by extracting the four-character ICD-10 code (`DIAG1_4`) and applying lexicographic range filters:

```sql
# SQL
WHERE (DIAG1_4 BETWEEN 'S000' AND 'S999')
   OR (DIAG1_4 BETWEEN 'T000' AND 'T149')
```

Assumption: This strategy captures inpatient admissions whose principal reason for hospitalization was coded as trauma. It does not capture cases where trauma appears only in secondary diagnoses.

Harmonizing region/comuna, sex, and age Because DEIS fields can contain numeric codes, text labels, missing markers (`'*'`), or blanks, the SQL layer standardizes them as follows.

Region of residence (`REGION_RESIDENCIA`)

- If numeric, the value is left-padded to 2 digits (e.g., `1` becomes `01`).
- Missing/blank values are retained as `'*'` in the initial recode step, then set to `NA` later in R.

Commune (municipality) of residence (`COMUNA_RESIDENCIA`)

- Blank or missing values are mapped to `'*'` initially; preserved for later cleaning decisions.

Sex (sexo_std)

- Mapped to male / female using either numeric codes (1, 2) or Spanish labels (HOMBRE, MUJER).
- All other values are mapped to unknown.

Age

- GRUPO_EDAD is parsed to obtain an approximate minimum age (age_min) using regex extraction of the first integer.
- Infant categories expressed in days/months or "MENOR …" are assigned age_min = 0.

The analysis uses age categories derived from age_min:

Variable	Categories
edad_cat	0 to 9, 10 to 18, 19 to 44, 45 to 59, 60 or more, unknown

Assumption: The DEIS age-group field is treated as a string containing sufficient information to recover a minimum-age proxy. This is a pragmatic harmonization step to support stratified descriptive analyses.

CMFt classification (cmf_group, is_cmf) CMFt flags are created using the predefined ICD-10 sets applied to the primary diagnosis (DIAG1_4):

- cmf_group = "soft" if DIAG1_4 is in CMF_SOFT_SET
- cmf_group = "hard" if DIAG1_4 is in CMF_HARD_SET
- is_cmf = 1 if DIAG1_4 is in CMF_SOFT_SET or CMF_HARD_SET, else 0

This yields a discharge-level CMFt indicator suitable for computing:

- CMFt burden within all hospitalized trauma (n_cmf / n_trauma)
- Separate trends for soft-tissue injuries vs facial fractures.

Encoding fallback for robustness DEIS files may differ in character encoding across years. The wrapper read_year_trauma() attempts multiple encodings (default: latin-1, then utf-8) and returns the first successful import. If both fail, the last error is raised to avoid silent data loss.

Build trauma_base (2001–2024) and QA hooks This section loops across all study years, extracts the standardized trauma cohort for each year, and concatenates them into a single analytic dataset.

Yearly ingestion and concatenation For each year from 2001 to 2024:

- Build the file path with file_for_year(y).
- Import via SQL using read_year_trauma() (with encoding fallback).
- Store the resulting year-specific data frame in a list.
- Concatenate all years into trauma_base and sort by discharge year.

A progress bar is displayed during ingestion, and a year-level log message prints:

- year

- encoding used
- number of extracted trauma rows
- number of CMFt rows (`sum(is_cmf == 1)`)

This log is a first-pass operational QA check to identify discontinuities in file parsing or case definitions.

Minimal hygiene and derived burden variables After concatenation, we apply minimal post-processing:

- Region cleaning: values such as `"99"`, `"EXTRANJERO"`, `"NULL"`, `"*"`, and empty strings are treated as missing (`NA`).
- Length of stay (LOS):
  - `los = DIAS_ESTADA` if non-missing and non-negative; otherwise `NA`.
  - Binary burden proxies:
    * `los_ge5 = 1` if `los >= 5`
    * `los_ge7 = 1` if `los >= 7`

These derived variables support later hospital-burden analyses (e.g., prolonged admissions).

Quick QA summary (`qa_year`) A compact year-level QA table is computed to monitor:

- `n`: total number of trauma discharges in that year.
- `pct_diag2_missing`: proportion of records with missing/blank `DIAG2` (external cause), highlighting etiology coding completeness.
- `pct_cmf`: proportion of trauma discharges flagged as CMFt.
- `deaths_cond2`: number of discharges with `CONDICION_EGRESO == "2"` (used here as an in-hospital death flag).

This QA object is printed for an immediate sanity check of early years before proceeding.

Etiology groups (`DIAG2`) and CMFt-only analytic subset After building `trauma_base`, etiology variables are appended using the previously defined mapping function:

```
trauma_base <- trauma_base %>%
  add_etiology_groups()
```

A CMFt-only analytic subset is then created:

- `cmf_base`: `trauma_base` filtered to `is_cmf == 1`.
- `ext_cause_6grp` is explicitly coerced so that missing/blank values become a dedicated category (`"No especificada/otras"`), enabling:
  - explicit reporting of missing external-cause information, and
  - consistent factor levels for plots and stratified summaries.

Code:

```
con <- dbConnect(
  duckdb::duckdb(),
  dbdir = file.path(PROJECT_DIR, "deis.duckdb"),
  read_only = FALSE
)
on.exit(dbDisconnect(con, shutdown = TRUE), add = TRUE)


sql_extract_trauma_year <- function(path, enc, year_expected, soft4, hard4) {
  path_sql <- gsub("'", "''", path)
  year_expected <- as.integer(year_expected)

  soft_sql <- paste(sprintf("'%s'", soft4), collapse = ",")
  hard_sql <- paste(sprintf("'%s'", hard4), collapse = ",")

  sprintf("
    WITH raw AS (
      SELECT
        coalesce(
          try_cast(regexp_extract(trim(ANO_EGRESO), '([0-9]{4})', 1) AS INTEGER),
          %d
        ) AS ANO_EGRESO,

        upper(trim(SEXO)) AS SEXO_TXT,
        upper(trim(GRUPO_EDAD)) AS AGE_TXT,
        trim(COMUNA_RESIDENCIA) AS COMUNA_RAW,
        trim(REGION_RESIDENCIA) AS REGION_RAW,

        upper(trim(substr(DIAG1,1,4))) AS DIAG1_4,
        upper(trim(substr(DIAG2,1,4))) AS DIAG2_4,

        try_cast(trim(DIAS_ESTADA) AS INTEGER) AS DIAS_ESTADA,
        trim(CONDICION_EGRESO) AS CONDICION_EGRESO

      FROM read_csv_auto('%s',
        delim=';',
        header=true,
        all_varchar=true,
        encoding='%s',
        strict_mode=false,
        ignore_errors=true,
        null_padding=true,
        max_line_size=10000000,
        sample_size=-1
      )
    ),
    trauma AS (
      SELECT *
      FROM raw
      WHERE
        (DIAG1_4 BETWEEN 'S000' AND 'S999')
        OR
        (DIAG1_4 BETWEEN 'T000' AND 'T149')
```

```
),
recode AS (
  SELECT
    ANO_EGRESO,

    CASE
      WHEN REGION_RAW IS NULL OR REGION_RAW = '' THEN '*'
      WHEN REGION_RAW = '*' THEN '*'
      WHEN regexp_matches(REGION_RAW, '^[0-9]+$') THEN lpad(REGION_RAW, 2, '0')
      ELSE REGION_RAW
    END AS REGION_RESIDENCIA,

    CASE
      WHEN COMUNA_RAW IS NULL OR COMUNA_RAW = '' THEN '*'
      WHEN COMUNA_RAW = '*' THEN '*'
      ELSE COMUNA_RAW
    END AS COMUNA_RESIDENCIA,

    CASE
      WHEN SEXO_TXT IN ('1','HOMBRE','MASCULINO') THEN 'male'
      WHEN SEXO_TXT IN ('2','MUJER','FEMENINO')   THEN 'female'
      ELSE 'unknown'
    END AS sexo_std,

    CASE
      WHEN AGE_TXT IS NULL OR AGE_TXT = '' OR AGE_TXT = '*' THEN NULL
      WHEN AGE_TXT LIKE 'MENOR%%' THEN 0
      WHEN AGE_TXT LIKE '%%DIAS%%' THEN 0
      WHEN AGE_TXT LIKE '%%MES%%'  THEN 0
      ELSE try_cast(regexp_extract(AGE_TXT, '([0-9]+)', 1) AS INTEGER)
    END AS age_min,

    DIAG1_4,
    DIAG2_4,
    DIAS_ESTADA,
    CONDICION_EGRESO
  FROM trauma
)
SELECT
  ANO_EGRESO,
  sexo_std,
  CASE
    WHEN age_min IS NULL OR age_min < 0 THEN 'unknown'
    WHEN age_min <= 9 THEN '0 to 9'
    WHEN age_min BETWEEN 10 AND 18 THEN '10 to 18'
    WHEN age_min BETWEEN 19 AND 44 THEN '19 to 44'
    WHEN age_min BETWEEN 45 AND 59 THEN '45 to 59'
    WHEN age_min >= 60 THEN '60 or more'
    ELSE 'unknown'
  END AS edad_cat,
  REGION_RESIDENCIA,
  COMUNA_RESIDENCIA,
  DIAG1_4 AS DIAG1,
```

```
      DIAG2_4 AS DIAG2,
      DIAS_ESTADA,
      CONDICION_EGRESO,
      CASE
        WHEN DIAG1_4 IN (%s) THEN 'soft'
        WHEN DIAG1_4 IN (%s) THEN 'hard'
        ELSE NULL
      END AS cmf_group,
      CASE
        WHEN DIAG1_4 IN (%s) OR DIAG1_4 IN (%s) THEN 1
        ELSE 0
      END AS is_cmf
    FROM recode
  ", year_expected, path_sql, enc, soft_sql, hard_sql, soft_sql, hard_sql)
}

read_year_trauma <- function(con, fpath, year_expected, soft_set, hard_set,
                             encodings = c("latin-1", "utf-8")) {
  last_err <- NULL
  for (enc in encodings) {
    q <- sql_extract_trauma_year(fpath, enc, year_expected, soft_set, hard_set)
    out <- tryCatch(DBI::dbGetQuery(con, q), error = function(e) { last_err <<- e; NULL })
    if (!is.null(out)) return(list(data = out, encoding = enc))
  }
  stop(last_err)
}


all_parts <- vector("list", length(YEARS))
names(all_parts) <- as.character(YEARS)

cli::cli_progress_bar("Building trauma_base (S00-S99 + T00-T14)", total = length(YEARS))

for (i in seq_along(YEARS)) {
  y <- YEARS[i]
  fpath <- file_for_year(y)

  res <- read_year_trauma(con, fpath, y, CMF_SOFT_SET, CMF_HARD_SET)
  df  <- res$data

  cli::cli_alert_success("Year {y} | Enc={res$encoding} | Rows={nrow(df)} | CMF={sum(df$is_cmf==1)}")
  cli::cli_progress_update(set = i)

  all_parts[[i]] <- df
}
cli::cli_progress_done()

trauma_base <- bind_rows(all_parts) %>%
  arrange(ANO_EGRESO)


trauma_base <- trauma_base %>%
  mutate(
```

```r
    REGION_RESIDENCIA = case_when(
      REGION_RESIDENCIA %in% c("99", "EXTRANJERO", "Extranjero", "NULL", "*", "") ~ NA_character_,
      TRUE ~ REGION_RESIDENCIA
    ),
    # LOS derived vars (hospital burden)
    los = if_else(DIAS_ESTADA < 0 | is.na(DIAS_ESTADA), NA_integer_, DIAS_ESTADA),
    los_ge5 = if_else(!is.na(los) & los >= 5, 1L, 0L),
    los_ge7 = if_else(!is.na(los) & los >= 7, 1L, 0L)
  )


qa_year <- trauma_base %>%
  group_by(ANO_EGRESO) %>%
  summarise(
    n = n(),
    pct_diag2_missing = mean(is.na(DIAG2) | DIAG2 == "", na.rm = TRUE),
    pct_cmf = mean(is_cmf == 1),
    deaths_cond2 = sum(trimws(CONDICION_EGRESO) == "2", na.rm = TRUE),
    .groups = "drop"
  )
print(head(qa_year, 10))


trauma_base <- trauma_base %>%
  add_etiology_groups()

cmf_base <- trauma_base %>%
  filter(is_cmf == 1) %>%
  mutate(
    ext_cause_6grp = if_else(
      is.na(ext_cause_6grp) | ext_cause_6grp == "",
      "No especificada/otras",
      as.character(ext_cause_6grp)
    ),
    ext_cause_6grp = factor(ext_cause_6grp)
  )
```

4. Data analysis

National rates of CMFt (per 1,000 trauma discharges)

This section estimates **national CMF trauma rates within hospitalized trauma**, expressed as **events per 1,000 trauma discharges**, and evaluates **longitudinal changes** using interrupted time-series (ITS) regression. The workflow proceeds in three steps: (i) annual aggregation, (ii) count-based ITS modeling with an offset for exposure (trauma discharges), and (iii) visualization of observed vs fitted rates with 95% confidence bands.

a) Count ITS for rates using an offset We define a generic helper, `fit_its_count_rate()`, that fits an ITS model to an annual count outcome while accounting for changes in the denominator (annual trauma discharges). The function:

1. Adds ITS covariates using `make_its_vars()`:

- `t`: centered time index from the first observed year
- `covid`: indicator for pandemic years
- `post`: indicator for post-acute period starting in `POST_YEAR`
- `t_post`: post-period slope term (0 pre-`POST_YEAR`)

2. Creates a count outcome and denominator:
   - `y = df[[count_col]]`
   - `denom = df[[denom_col]]` (default: `n_trauma`)
   - `log_denom = log(denom)` as the **offset** term
3. Fits either:
   - **Negative binomial** (`MASS::glm.nb`) to handle overdispersion, or
   - **Poisson** (`glm(..., family = poisson(link="log"))`) as the baseline count model

Model form (conceptually): - Outcome: annual count `y` - Linear predictor includes ITS terms (`t`, `covid`, `post`, `t_post`) - Offset: `log(denom)` so coefficients represent changes in the **rate** rather than raw counts

b) Predictions on the rate scale (per 1,000) Predictions are generated on the **link scale** and then exponentiated to obtain fitted expected counts: - `mu_hat = exp(fitted_link)` - 95% confidence bands on the count scale: - `mu_lo = exp(fitted_link - 1.96 * SE_link)` - `mu_hi = exp(fitted_link + 1.96 * SE_link)`

These are converted to rates per 1,000 trauma discharges: - Observed rate: - `rate_obs_per_1000 = 1000 * (y_obs / denom)` - Fitted rate and 95% CI: - `rate_fit_per_1000 = 1000 * (mu_hat / denom)` - `rate_lo_per_1000  = 1000 * (mu_lo  / denom)` - `rate_hi_per_1000  = 1000 * (mu_hi  / denom)`

The function returns: - `model`: fitted GLM/GLM-NB object - `pred`: year-level observed/fitted rates with CI - metadata: `outcome`, `family`

c) Annual aggregation for national ITS Annual national totals are computed from `trauma_base`:

- `n_trauma`: total trauma discharges (denominator)
- `n_cmf`: CMF discharges (soft + hard)
- `n_hard`: CMF hard (fractures)
- `n_soft`: CMF soft (soft-tissue / non-fracture CMF)

These provide three ITS outcomes: - total CMF rate within trauma (`n_cmf`) - hard CMF rate within trauma (`n_hard`) - soft CMF rate within trauma (`n_soft`)

All three series are fit using negative binomial ITS by default (`model = "negbin"`), which is typically preferable for administrative count data with overdispersion.

Optional robust standard errors are computed via `coeftest_robust(model)` to support reporting in tables.

Overdispersion is assessed using the Pearson dispersion statistic: - compute Pearson residuals (`type="pearson"`) - `dispersion = sum(rp^2) / df_residual`

Values substantially above 1 suggest overdispersion, motivating negative binomial over Poisson.

d) Visualization: national rates ITS figure A combined dataset of predictions (`pred_rates_all`) is assembled for the three series (CMF total, hard, soft). The figure uses:

- **Points**: observed annual rates (`rate_obs_per_1000`)
- **Line**: fitted ITS rates (`rate_fit_per_1000`)

- **Ribbon**: 95% CI (`rate_lo_per_1000`, `rate_hi_per_1000`)
- **Vertical dotted lines**: key boundaries (default: 2019 and 2022)

Interpretation: - Level changes during COVID years are captured by the `covid` indicator. - A post-2022 level and slope change are captured by `post` and `t_post`.

```
fit_its_count_rate <- function(df, count_col, denom_col = "n_trauma",
                               year_col = "ANO_EGRESO",
                               model = c("poisson", "negbin"),
                               post_year = POST_YEAR, covid_years = COVID_YEARS) {
  model <- match.arg(model)

  dat <- df %>%
    make_its_vars(year_col = year_col, post_year = post_year, covid_years = covid_years) %>%
    mutate(
      y = .data[[count_col]],
      denom = .data[[denom_col]],
      log_denom = log(denom)
    )

  # Fit
  if (model == "negbin") {
    m <- MASS::glm.nb(y ~ t + covid + post + t_post + offset(log_denom), data = dat)
  } else {
    m <- glm(y ~ t + covid + post + t_post + offset(log_denom),
             family = poisson(link = "log"), data = dat)
  }

  # Predict: expected counts then convert to rate per 1000
  pred <- broom::augment(m, newdata = dat, type.predict = "link", se_fit = TRUE) %>%
    transmute(
      !!year_col := dat[[year_col]],
      denom = dat$denom,
      y_obs = dat$y,
      # predicted mean on response scale:
      mu_hat = exp(.fitted),
      mu_lo  = exp(.fitted - 1.96 * .se.fit),
      mu_hi  = exp(.fitted + 1.96 * .se.fit),
      rate_obs_per_1000 = 1000 * (y_obs / denom),
      rate_fit_per_1000 = 1000 * (mu_hat / denom),
      rate_lo_per_1000  = 1000 * (mu_lo  / denom),
      rate_hi_per_1000  = 1000 * (mu_hi  / denom)
    )

  list(model = m, pred = pred, outcome = count_col, family = model)
}

agg_year <- trauma_base %>%
  dplyr::group_by(ANO_EGRESO) %>%
  dplyr::summarise(
    n_trauma = dplyr::n(),
    n_cmf = sum(is_cmf == 1),
    n_hard = sum(cmf_group == "hard", na.rm = TRUE),
    n_soft = sum(cmf_group == "soft", na.rm = TRUE),
```

```r
    .groups = "drop"
  )

its_rate_total_nb <- fit_its_count_rate(agg_year, count_col = "n_cmf",  model = "negbin")
its_rate_hard_nb  <- fit_its_count_rate(agg_year, count_col = "n_hard", model = "negbin")
its_rate_soft_nb  <- fit_its_count_rate(agg_year, count_col = "n_soft", model = "negbin")

# (Opcional) robust SE para tabla
print(coeftest_robust(its_rate_total_nb$model))
print(coeftest_robust(its_rate_hard_nb$model))
print(coeftest_robust(its_rate_soft_nb$model))

pred_rates_all <- dplyr::bind_rows(
  its_rate_total_nb$pred %>% mutate(series = "CMF total"),
  its_rate_hard_nb$pred  %>% mutate(series = "CMF hard"),
  its_rate_soft_nb$pred  %>% mutate(series = "CMF soft")
) %>%
  mutate(series = factor(series, levels = c("CMF total", "CMF hard", "CMF soft")))

fig_its_rates <- ggplot(pred_rates_all, aes(x = ANO_EGRESO)) +
  geom_ribbon(aes(ymin = rate_lo_per_1000, ymax = rate_hi_per_1000, fill = series),
              alpha = 0.15, color = NA) +
  geom_line(aes(y = rate_fit_per_1000, color = series), linewidth = 1.05) +
  geom_point(aes(y = rate_obs_per_1000, color = series), size = 1.7, alpha = 0.75) +
  geom_vline(xintercept = c(2019, 2022), linetype = "dotted") +
  labs(
    x = "Year",
    y = "Rate per 1,000 trauma discharges",
    title = "Interrupted time series: national CMF trauma rates within hospitalized trauma",
    subtitle = "Poisson count ITS with log(n_trauma) offset; lines = fitted rates, points = observed ra
    color = "Series",
    fill  = "Series"
  ) +
  theme_minimal(base_size = 13) +
  theme(legend.position = "right")

print(fig_its_rates)

check_overdispersion <- function(m) {
  rp <- residuals(m, type = "pearson")
  disp <- sum(rp^2) / df.residual(m)
  disp
}

cat("Dispersion total:", check_overdispersion(its_rate_total_nb$model), "\n")
cat("Dispersion hard :", check_overdispersion(its_rate_hard_nb$model), "\n")
cat("Dispersion soft :", check_overdispersion(its_rate_soft_nb$model), "\n")
```

This section evaluates longitudinal changes in **hospital burden** measures, focusing on **length of stay (LOS)** and **prolonged hospitalization**.

e) Mean LOS (overall CMF) by injury type (hard vs soft) A year-by-type summary dataset is created among CMF admissions only:

- Filter: `is_cmf == 1` and `cmf_group` in `{hard, soft}`
- Aggregate by year and type:
  - `n_cmf`: number of CMF admissions
  - `n_los`: number with non-missing LOS
  - `mean_los`, `sd_los`

Records with no valid LOS (`n_los == 0`) are removed to avoid implicit dropping by `lm()` without visibility.

ITS model with type interactions `fit_its_lm_bytype()` fits a linear ITS model with **interaction terms** to compare hard vs soft trajectories:

- Outcome: `mean_los` (continuous)
- Model:
  - baseline + ITS terms (`t`, `covid`, `post`, `t_post`)
  - plus interactions with type (`cmf_group`), allowing:
    - different baseline levels (hard vs soft)
    - different secular trend
    - different COVID level shift
    - different post-2022 level change
    - different post-2022 slope change

Predictions include 95% confidence intervals: - `y_fit = fitted` - `y_lo`, `y_hi` computed as `fitted ± 1.96 * SE(fitted)`

A plot analogous to Section 07 is produced: - points: observed mean LOS - line: fitted ITS mean LOS - ribbon: 95% CI - dotted vertical lines: 2019 and 2022

```
agg_year_los_bytype <- trauma_base %>%
  dplyr::filter(
    is_cmf == 1,
    cmf_group %in% c("hard", "soft")
  ) %>%
  dplyr::group_by(ANO_EGRESO, cmf_group) %>%
  dplyr::summarise(
    n_cmf    = dplyr::n(),
    n_los    = sum(!is.na(los)),
    mean_los = mean(los, na.rm = TRUE),
    sd_los   = sd(los, na.rm = TRUE),
    .groups  = "drop"
  ) %>%
  dplyr::mutate(
    cmf_group = factor(cmf_group, levels = c("soft", "hard")) # baseline = soft
  ) %>%
  dplyr::arrange(ANO_EGRESO, cmf_group) %>%
  dplyr::filter(n_los > 0, !is.na(mean_los))


fit_its_lm_bytype <- function(df, y, transform = c("none", "logit"),
                              year_col = "ANO_EGRESO",
                              type_col = "cmf_group",
                              post_year = POST_YEAR,
                              covid_years = COVID_YEARS) {
```

```r
  transform <- match.arg(transform)

  dat <- df %>%
    make_its_vars(year_col = year_col) %>%
    mutate(
      .y_raw = .data[[y]],
      .type  = factor(.data[[type_col]])
    )

  if (transform == "logit") {
    dat <- dat %>% mutate(.y = qlogis(clamp01(.y_raw)))
  } else {
    dat <- dat %>% mutate(.y = .y_raw)
  }

  # ITS with type interactions:
  # baseline + time + covid + post + slope post
  # plus differences for hard vs soft in each component
  m <- lm(.y ~ .type * (t + covid + post + t_post), data = dat)

  # predictions + 95% CI
  pred <- broom::augment(m, newdata = dat, se_fit = TRUE) %>%
    transmute(
      !!year_col := .data[[year_col]],
      !!type_col := dat$.type,
      y_obs = dat$.y_raw,
      y_fit = .fitted,
      y_lo  = .fitted - 1.96 * .se.fit,
      y_hi  = .fitted + 1.96 * .se.fit
    )

  if (transform == "logit") {
    pred <- pred %>%
      mutate(
        y_fit = plogis(y_fit),
        y_lo  = plogis(y_lo),
        y_hi  = plogis(y_hi)
      )
  }

  list(model = m, pred = pred, transform = transform, outcome = y)
}

its_meanlos_bytype <- fit_its_lm_bytype(agg_year_los_bytype, y = "mean_los", transform = "none")

print(summary(its_meanlos_bytype$model))
print(coeftest_robust(its_meanlos_bytype$model))

pred_meanlos_bytype <- its_meanlos_bytype$pred %>%
  mutate(
    cmf_group = factor(cmf_group, levels = c("soft","hard"))
  )
```

```
fig_its_meanlos_bytype <- ggplot(pred_meanlos_bytype, aes(x = ANO_EGRESO)) +
  geom_ribbon(aes(ymin = y_lo, ymax = y_hi, fill = cmf_group), alpha = 0.18, color = NA) +
  geom_line(aes(y = y_fit, color = cmf_group), linewidth = 1.05) +
  geom_point(aes(y = y_obs, color = cmf_group), size = 1.7, alpha = 0.75) +
  geom_vline(xintercept = c(2019, 2022), linetype = "dotted") +
  labs(
    x = "Year",
    y = "Mean LOS (days)",
    title = "Interrupted time series: mean LOS after CMF trauma by injury type",
    subtitle = "Hard vs soft modeled jointly with interaction terms (type × time periods)",
    color = "CMF type",
    fill  = "CMF type"
  ) +
  theme_minimal(base_size = 13) +
  theme(legend.position = "right")

print(fig_its_meanlos_bytype)
```

f) Proportion LOS 5 among hard CMF (logit scale) To model a bounded proportion, hard CMF admissions are aggregated by year:

- `p_los_ge5 = mean(los_ge5)` (0–1 proportion)
- `n_los_ge5`: count of admissions with LOS 5

`fit_its_lm(..., transform="logit")` is then used: - outcome is transformed to log-odds via `qlogis(clamp01(p))` - OLS ITS is fit on the linear predictor scale - predictions and CIs are computed on the linear scale and back-transformed with `plogis()`

The output figure (`plot_its`) presents: - points: observed annual proportion - line: fitted proportion - ribbon: 95% CI on the proportion scale - optional percent formatting (`percent = TRUE`)

```
cmf_hard_year_los <- cmf_base %>%
  dplyr::filter(cmf_group == "hard") %>%
  dplyr::group_by(ANO_EGRESO) %>%
  dplyr::summarise(
    n_hard = dplyr::n(),
    n_los_ge5 = sum(los_ge5, na.rm = TRUE),
    p_los_ge5 = mean(los_ge5, na.rm = TRUE),    # proporción 0-1
    .groups = "drop"
  )

print(head(cmf_hard_year_los, 10))

its_plos5_hard <- fit_its_lm(cmf_hard_year_los, y = "p_los_ge5", transform = "logit")

print(summary(its_plos5_hard$model))
print(coeftest_robust(its_plos5_hard$model))

fig_its_plos5_hard <- plot_its(
  its_plos5_hard$pred,
  ylab = "Proportion of hard CMF admissions with LOS  5 days",
  title = "Interrupted time series: prolonged hospitalization after hard CMF trauma",
```

```
  subtitle = "Outcome: p(LOS  5 days) among CMF hard admissions only",
  percent = TRUE
)

print(fig_its_plos5_hard)
```

g) Etiology-specific LOS burden over time Etiology groups are ordered by overall mean LOS to stabilize legend ordering and improve interpretability.

Year-by-etiology summaries are computed among CMF admissions with known etiology:

- `median_los` and `mean_los`
- `p_los_ge7` (proportion LOS 7 days)
- `n` admissions

A time-series plot is generated for median LOS by etiology, with dotted reference lines at 2019 and 2022.

```
etiol_order <- cmf_base %>%
  filter(!is.na(ext_cause_6grp)) %>%
  group_by(ext_cause_6grp) %>%
  summarise(
    n = n(),
    mean_los = mean(los, na.rm = TRUE),
    .groups = "drop"
  ) %>%
  arrange(desc(mean_los)) %>%
  pull(ext_cause_6grp)

agg_year_los_etiol_cmf <- cmf_base %>%
  filter(!is.na(ext_cause_6grp)) %>%
  group_by(ANO_EGRESO, ext_cause_6grp) %>%
  summarise(
    n = n(),
    mean_los = mean(los, na.rm = TRUE),
    median_los = median(los, na.rm = TRUE),
    p_los_ge7 = mean(los_ge7, na.rm = TRUE),
    .groups = "drop"
  ) %>%
  mutate(ext_cause_6grp = factor(ext_cause_6grp, levels = etiol_order))

fig_los_etiol <- ggplot(agg_year_los_etiol_cmf, aes(x = ANO_EGRESO, y = mean_los, group = ext_cause_6gr
  geom_line(linewidth = 1.0) +
  geom_point(size = 1.5, alpha = 0.85) +
  geom_vline(xintercept = c(2019, 2022), linetype = "dotted") +
  coord_cartesian(ylim = c(0, 15))+
  labs(
    x = "Year",
    y = "Mean LOS (days)",
    title = "Hospital burden of CMF trauma by etiology",
    subtitle = "Outcome: mean LOS, CMF admissions",
    color = "Etiology (6 groups)"
  ) +
```

```
  theme_minimal(base_size = 13) +
  theme(legend.position = "right")
print(fig_los_etiol)
```

h) Etiology composition (stacked area) among known external causes To describe composition conditional on coding completeness:

1. Define the 6 canonical etiology levels (`etiol_6_levels`) and a dedicated missingness level (`"No especificada/otras"`).
2. Count admissions by year and etiology (`etiol_counts_year`).
3. Compute year totals, including:
   - `n_cmf_total`
   - `n_known`
   - `n_missing`
   - `pct_missing`

The stacked area plot (`fig_mix_main`) is computed **among known external causes only**: - complete missing combinations as zeros (`tidyr::complete`) - compute percentages within known (`pct = 100 * n / denom_known`)

Interpretation: - The plot shows **etiology mix conditional on DIAG2 being present and classifiable**, while `etiol_year_totals` provides a parallel QA view of missingness over time.

```
etiol_6_levels <- c(
  "Unspecified mechanism of accidental injury",
  "Low-energy accidental injuries",
  "Transport-related accidental injuries (high-energy)",
  "Interpersonal violence",
  "Non-accidental injuries or complex external causes",
  "Other rare external causes"
)

etiol_missing_level <- "No especificada/otras"

etiol_counts_year <- cmf_base %>%
  dplyr::mutate(
    ext6 = as.character(ext_cause_6grp),
    ext6 = dplyr::if_else(is.na(ext6) | ext6 == "", etiol_missing_level, ext6),
    ext6 = dplyr::if_else(!(ext6 %in% c(etiol_6_levels, etiol_missing_level)), etiol_missing_level, ext6
  ) %>%
  dplyr::count(ANO_EGRESO, ext6, name = "n")

etiol_year_totals <- etiol_counts_year %>%
  dplyr::group_by(ANO_EGRESO) %>%
  dplyr::summarise(
    n_cmf_total = sum(n),
    n_known = sum(n[ext6 %in% etiol_6_levels]),
    n_missing = sum(n[ext6 == etiol_missing_level]),
    pct_missing = 100 * n_missing / n_cmf_total,
    .groups = "drop"
  )
```

```r
print(head(etiol_year_totals, 10))

etiol_mix_year_main <- etiol_counts_year %>%
  dplyr::filter(ext6 %in% etiol_6_levels) %>%
  tidyr::complete(
    ANO_EGRESO,
    ext6 = etiol_6_levels,
    fill = list(n = 0)
  ) %>%
  dplyr::group_by(ANO_EGRESO) %>%
  dplyr::mutate(
    denom_known = sum(n),
    pct = dplyr::if_else(denom_known > 0, 100 * n / denom_known, NA_real_)
  ) %>%
  dplyr::ungroup() %>%
  dplyr::mutate(ext6 = factor(ext6, levels = etiol_6_levels))

fig_mix_main <- ggplot(etiol_mix_year_main, aes(x = ANO_EGRESO, y = pct, fill = ext6)) +
  geom_area(alpha = 0.9, color = "white", linewidth = 0.2) +
  scale_fill_brewer(palette = "Set2")+
  geom_vline(xintercept = c(2019, 2022), linetype = "dotted") +
  scale_y_continuous(labels = scales::percent_format(scale = 1)) +
  labs(
    x = "Year",
    y = "Proportion of CMF admissions (%)",
    title = "Etiological composition of craniomaxillofacial trauma",
    subtitle = "Percentages computed among admissions with known external cause (6-group classification
    fill = "Etiology (6 groups)"
  ) +
  theme_minimal(base_size = 12) +
  theme(legend.position = "right")

print(fig_mix_main)
```

i) EPI: evolution of CMF HARD diagnoses over time (composition within CMFt only) This section evaluates how the **diagnostic mix of hard CMF injuries** (fracture subtypes) evolves over time, restricting to CMF hard admissions only.

Diagnostic labeling and safety checks A label map (`hard_diag_labels`) assigns human-readable labels to each ICD-10 hard CMF code.

A safety check verifies that any record labeled as hard CMF has a `DIAG1` code contained in `CMF_HARD_SET`. Any codes outside the set are printed as a warning for QA.

Annual diagnostic composition within hard CMF

1. Count `n` by year × `DIAG1` among hard CMF admissions.
2. Normalize within year to obtain:
    - `prop_within_hard = n / sum(n)`
    - `pct_within_hard = 100 * prop_within_hard`
3. QA check confirms that `sum(prop_within_hard) == 1` each year.

A stacked area plot visualizes the within-hard diagnostic composition over time: - y-axis: proportion within hard CMF (sums to 1 each year) - dotted vertical lines: 2019 and 2022

```
hard_diag_labels <- c(
  "S020" = "S020 - Fracture of vault of skull",
  "S022" = "S022 - Nasal bones fracture",
  "S023" = "S023 - Orbital floor fracture",
  "S024" = "S024 - Malar & maxillary fracture",
  "S026" = "S026 - Mandibular fracture",
  "S027" = "S027 - Multiple skull and facial bones fractures",
  "S028" = "S028 - Other specified skull and facial bones fractures",
  "S029" = "S029 - Unspecified facial fracture"
)


hard_outside_set <- cmf_base %>%
  dplyr::filter(cmf_group == "hard", !is.na(DIAG1), DIAG1 != "") %>%
  dplyr::distinct(DIAG1) %>%
  dplyr::filter(!DIAG1 %in% CMF_HARD_SET)

if (nrow(hard_outside_set) > 0) {
  message("WARNING: Found 'hard' DIAG1 outside CMF_HARD_SET:")
  print(hard_outside_set)
} else {
  message("OK: All hard DIAG1 are within CMF_HARD_SET.")
}


cmf_hard_year_diag <- cmf_base %>%
  dplyr::filter(
    cmf_group == "hard",
    !is.na(DIAG1),
    DIAG1 != "",
    DIAG1 %in% CMF_HARD_SET
  ) %>%
  dplyr::count(ANO_EGRESO, DIAG1, name = "n")


cmf_hard_mix_year <- cmf_hard_year_diag %>%
  dplyr::group_by(ANO_EGRESO) %>%
  dplyr::mutate(
    prop_within_hard = n / sum(n),
    pct_within_hard  = 100 * prop_within_hard
  ) %>%
  dplyr::ungroup() %>%
  dplyr::mutate(
    DIAG1 = factor(DIAG1, levels = CMF_HARD_SET),
    DIAG1_label = dplyr::recode(as.character(DIAG1), !!!hard_diag_labels),
    DIAG1_label = factor(DIAG1_label, levels = unname(hard_diag_labels))
  )


qa_sums <- cmf_hard_mix_year %>%
  dplyr::group_by(ANO_EGRESO) %>%
```

```r
  dplyr::summarise(sum_prop = sum(prop_within_hard), .groups = "drop")
print(qa_sums)


fig_hard_mix_within_hard <- ggplot(
  cmf_hard_mix_year,
  aes(x = ANO_EGRESO, y = prop_within_hard, fill = DIAG1_label)
) +
  geom_area(alpha = 0.9, color = "white", linewidth = 0.2) +
  scale_fill_brewer(palette = "Accent")+
  geom_vline(xintercept = c(2019, 2022), linetype = "dotted") +
  scale_y_continuous(labels = scales::percent_format(accuracy = 1)) +
  labs(
    x = "Year",
    y = "Composition within CMF hard (%)",
    title = "Evolution of CMF hard diagnosis mix over time",
    subtitle = "Composition among CMF hard admissions only (CIE-10 S02.2-S02.9 subset)",
    fill = "Hard diagnosis (CIE-10)"
  ) +
  theme_minimal(base_size = 12) +
  theme(legend.position = "right")

print(fig_hard_mix_within_hard)
```

j) Heat map: regional CMF rate within hospitalized trauma (mean 2001–2024) This section produces a regional map of CMF trauma rates within trauma admissions.

Considerations

1. Ensure region codes are 2-digit strings.
2. Aggregate by region × year:
    - `n_trauma`
    - `n_cmf`
    - `rate_cmf_per_1000 = 1000 * n_cmf / n_trauma`
3. Compute a region-level summary across all years using a weighted approach:
    - `w_rate_per_1000 = 1000 * (sum n_cmf) / (sum n_trauma)`

This yields a stable long-run rate per region, weighting years by annual trauma volume.

4. Join the regional summary to a spatial polygon layer (`chilemapas::generar_regiones()`), matching by region code.

Map output A choropleth map displays `w_rate_per_1000` using a log-scale fill transform to handle skewness, with missing regions shown as white.

Interpretation: - The map summarizes the **long-run regional CMF rate within hospitalized trauma**, rather than year-to-year volatility. - Because it is weighted by total trauma volume, small regions with low denominators are less likely to dominate due to unstable annual rates.

```r
tb_reg <- trauma_base %>%
  dplyr::filter(!is.na(REGION_RESIDENCIA)) %>%
  dplyr::mutate(REGION_RESIDENCIA = stringr::str_pad(REGION_RESIDENCIA, 2, pad = "0"))


reg_year <- tb_reg %>%
  dplyr::group_by(REGION_RESIDENCIA, ANO_EGRESO) %>%
  dplyr::summarise(
    n_trauma = dplyr::n(),
    n_cmf    = sum(is_cmf == 1, na.rm = TRUE),
    rate_cmf_per_1000 = 1000 * n_cmf / n_trauma,
    .groups = "drop"
  )

reg_mean_weighted <- reg_year %>%
  dplyr::group_by(REGION_RESIDENCIA) %>%
  dplyr::summarise(
    n_trauma_total = sum(n_trauma, na.rm = TRUE),
    n_cmf_total    = sum(n_cmf, na.rm = TRUE),
    w_rate_per_1000 = 1000 * n_cmf_total / n_trauma_total,
    .groups = "drop"
  )

reg_sf <- chilemapas::generar_regiones()

names(reg_sf)

map_df <- reg_sf %>%
  dplyr::mutate(
    REGION_RESIDENCIA = stringr::str_pad(as.character(codigo_region), 2, pad = "0")
  ) %>%
  dplyr::left_join(reg_mean_weighted, by = "REGION_RESIDENCIA")

ggplot(map_df) +
  geom_sf(aes(fill = w_rate_per_1000), color = "white", linewidth = 0.2) +
  scale_fill_gradient(
    low = "grey95",
    trans = "log10",
    high = "#33549F",
    na.value = "white",
    name = "Rate / 1,000"
  ) +
  labs(
    title = "CMF trauma rate within hospitalized trauma (mean 2001-2024)",
    subtitle = "Rate per 1,000 trauma discharges - simple mean of annual region-specific rates"
  ) +
  theme_minimal(base_size = 12)
```

Stratified trends and interrupted time-series panels (age group and sex)

These panels provide a descriptive and model-based view of how craniomaxillofacial trauma (CMFt) patterns vary by **age group** and **sex** across 2001–2024. We first plot **stratified annual rates** (per 1,000 hospitalized trauma discharges) to summarize long-run trends when aggregating either across sex (age-only facets) or

across age (sex-only facets). We then fit **stratum-specific interrupted time-series (ITS)** models for CMF **total**, **hard**, and **soft** injuries, and extend the approach to **mean length of stay (LOS)** among CMF admissions.

Key implementation details:

- **Strata and denominators:** Age groups follow the study categorization (`0-9`, `10-18`, `19-44`, `45-59`, `60+`). To keep denominators comparable across years, records with **unknown sex** and/or **unknown age category** are excluded where indicated in the filters.
- **Rate definition:** Annual rates are computed as `1000 × (CMF count / all trauma discharges)` within each stratum.
- **ITS for rates:** We fit segmented **count models** using a **log(n_trauma) offset** (default: negative binomial). If the negative binomial fit fails (e.g., sparse strata with many zeros), the code automatically falls back to **Poisson**.
- **ITS for mean LOS:** Mean LOS is modeled with a segmented **linear model** in strata with a minimum number of non-missing annual means (guardrail to avoid unstable fits).
- **Temporal markers:** Dotted vertical lines at **2019** (last full pre-COVID year) and **2022** (start of the post-acute period in our main ITS specification) are used to visually align panels with the national-level ITS breakpoints.

Finally, two stacked-area panels summarize within-stratum composition:

- **Diagnostic mix (CMF hard):** For bone fractures, we plot the **within-year, within-stratum proportion** of each ICD-10 primary diagnosis category (`DIAG1`).
- **Etiology mix (known causes):** Etiology composition is shown as **percentages among admissions with a known external cause** (six-group classification), by year and stratum.

```
age_levels <- c("0 to 9","10 to 18","19 to 44","45 to 59","60 or more")

agg_year_age <- trauma_base %>%
  dplyr::filter(
    !is.na(ANO_EGRESO),
    !is.na(edad_cat), edad_cat %in% age_levels,
    sexo_std %in% c("male","female") # opcional: para excluir unknown
  ) %>%
  dplyr::group_by(ANO_EGRESO, edad_cat) %>%
  dplyr::summarise(
    n_trauma = dplyr::n(),
    n_cmf    = sum(is_cmf == 1, na.rm = TRUE),
    n_hard   = sum(cmf_group == "hard", na.rm = TRUE),
    n_soft   = sum(cmf_group == "soft", na.rm = TRUE),
    .groups  = "drop"
  ) %>%
  dplyr::mutate(
    edad_cat = factor(edad_cat, levels = age_levels),
    rate_cmf_per_1000  = dplyr::if_else(n_trauma > 0, 1000 * n_cmf  / n_trauma, NA_real_),
    rate_hard_per_1000 = dplyr::if_else(n_trauma > 0, 1000 * n_hard / n_trauma, NA_real_),
    rate_soft_per_1000 = dplyr::if_else(n_trauma > 0, 1000 * n_soft / n_trauma, NA_real_)
  )

trend_age_long <- agg_year_age %>%
  tidyr::pivot_longer(
    cols = c(rate_cmf_per_1000, rate_hard_per_1000, rate_soft_per_1000),
```

```r
    names_to = "series",
    values_to = "rate_per_1000"
  ) %>%
  dplyr::mutate(
    series = dplyr::recode(
      series,
      rate_cmf_per_1000  = "CMF total",
      rate_hard_per_1000 = "CMF hard",
      rate_soft_per_1000 = "CMF soft"
    ),
    series = factor(series, levels = c("CMF total","CMF hard","CMF soft"))
  )

fig_trends_by_age <- ggplot(trend_age_long, aes(x = ANO_EGRESO, y = rate_per_1000, color = series)) +
  geom_line(linewidth = 1.0, alpha = 0.95) +
  geom_point(size = 1.3, alpha = 0.65) +
  geom_vline(xintercept = c(2019, 2022), linetype = "dotted") +
  facet_wrap(~ edad_cat, ncol = 3, scales = "free_y") +
  labs(
    x = "Year",
    y = "Rate per 1,000 trauma discharges",
    title = "CMF trauma trends by age group",
    subtitle = "Within hospitalized trauma; colors = CMF total/hard/soft (sex aggregated)",
    color = "Series"
  ) +
  theme_minimal(base_size = 12) +
  theme(legend.position = "bottom")

print(fig_trends_by_age)


agg_year_sex <- trauma_base %>%
  dplyr::filter(
    !is.na(ANO_EGRESO),
    sexo_std %in% c("male","female"),
    !is.na(edad_cat), edad_cat %in% age_levels
  ) %>%
  dplyr::group_by(ANO_EGRESO, sexo_std) %>%
  dplyr::summarise(
    n_trauma = dplyr::n(),
    n_cmf    = sum(is_cmf == 1, na.rm = TRUE),
    n_hard   = sum(cmf_group == "hard", na.rm = TRUE),
    n_soft   = sum(cmf_group == "soft", na.rm = TRUE),
    .groups  = "drop"
  ) %>%
  dplyr::mutate(
    sexo_std = factor(sexo_std, levels = c("male","female")),
    rate_cmf_per_1000  = dplyr::if_else(n_trauma > 0, 1000 * n_cmf  / n_trauma, NA_real_),
    rate_hard_per_1000 = dplyr::if_else(n_trauma > 0, 1000 * n_hard / n_trauma, NA_real_),
    rate_soft_per_1000 = dplyr::if_else(n_trauma > 0, 1000 * n_soft / n_trauma, NA_real_)
  )

trend_sex_long <- agg_year_sex %>%
```

```
  tidyr::pivot_longer(
    cols = c(rate_cmf_per_1000, rate_hard_per_1000, rate_soft_per_1000),
    names_to = "series",
    values_to = "rate_per_1000"
  ) %>%
  dplyr::mutate(
    series = dplyr::recode(
      series,
      rate_cmf_per_1000  = "CMF total",
      rate_hard_per_1000 = "CMF hard",
      rate_soft_per_1000 = "CMF soft"
    ),
    series = factor(series, levels = c("CMF total","CMF hard","CMF soft"))
  )

fig_trends_by_sex <- ggplot(trend_sex_long, aes(x = ANO_EGRESO, y = rate_per_1000, color = series)) +
  geom_line(linewidth = 1.0, alpha = 0.95) +
  geom_point(size = 1.3, alpha = 0.65) +
  geom_vline(xintercept = c(2019, 2022), linetype = "dotted") +
  facet_wrap(~ sexo_std, ncol = 1, scales = "free_y") +
  labs(
    x = "Year",
    y = "Rate per 1,000 trauma discharges",
    title = "CMF trauma trends by sex",
    subtitle = "Within hospitalized trauma; colors = CMF total/hard/soft (age aggregated)",
    color = "Series"
  ) +
  theme_minimal(base_size = 12) +
  theme(legend.position = "bottom")

print(fig_trends_by_sex)


# ============================================================
# 3) ITS por ESTRATO (EDAD × SEXO) para CMF total/hard/soft
#    -> NegBin con offset log(n_trauma)
# ============================================================

agg_year_sex_age <- trauma_base %>%
  dplyr::filter(
    !is.na(ANO_EGRESO),
    sexo_std %in% c("male","female"),
    !is.na(edad_cat), edad_cat %in% age_levels
  ) %>%
  dplyr::group_by(ANO_EGRESO, sexo_std, edad_cat) %>%
  dplyr::summarise(
    n_trauma = dplyr::n(),
    n_cmf    = sum(is_cmf == 1, na.rm = TRUE),
    n_hard   = sum(cmf_group == "hard", na.rm = TRUE),
    n_soft   = sum(cmf_group == "soft", na.rm = TRUE),
    .groups  = "drop"
  ) %>%
  dplyr::mutate(
```

```r
    sexo_std = factor(sexo_std, levels = c("male","female")),
    edad_cat = factor(edad_cat, levels = age_levels)
  )


safe_fit_its_rate <- function(df, count_col) {
  out <- tryCatch(
    fit_its_count_rate(df, count_col = count_col, denom_col = "n_trauma", model = "negbin"),
    error = function(e) NULL
  )
  if (is.null(out)) {
    out <- fit_its_count_rate(df, count_col = count_col, denom_col = "n_trauma", model = "poisson")
  }
  out
}

its_pred_strata <- agg_year_sex_age %>%
  dplyr::group_by(sexo_std, edad_cat) %>%
  dplyr::group_modify(~{
    df <- .x

    its_total <- safe_fit_its_rate(df, "n_cmf")
    its_hard  <- safe_fit_its_rate(df, "n_hard")
    its_soft  <- safe_fit_its_rate(df, "n_soft")

    dplyr::bind_rows(
      its_total$pred %>% dplyr::mutate(series = "CMF total"),
      its_hard$pred  %>% dplyr::mutate(series = "CMF hard"),
      its_soft$pred  %>% dplyr::mutate(series = "CMF soft")
    )
  }) %>%
  dplyr::ungroup() %>%
  dplyr::mutate(series = factor(series, levels = c("CMF total","CMF hard","CMF soft")))

fig_its_by_stratum <- ggplot(its_pred_strata, aes(x = ANO_EGRESO)) +
  geom_ribbon(aes(ymin = rate_lo_per_1000, ymax = rate_hi_per_1000, fill = series),
              alpha = 0.12, color = NA) +
  geom_line(aes(y = rate_fit_per_1000, color = series), linewidth = 1.0) +
  geom_point(aes(y = rate_obs_per_1000, color = series), size = 1.1, alpha = 0.6) +
  geom_vline(xintercept = c(2019, 2022), linetype = "dotted") +
  facet_grid(sexo_std ~ edad_cat, scales = "free_y") +
  labs(
    x = "Year",
    y = "Rate per 1,000 trauma discharges",
    title = "Interrupted time series by age group and sex strata",
    color = "Series",
    fill  = "Series"
  ) +
  theme_minimal(base_size = 11) +
  theme(legend.position = "bottom") +
  guides(fill = "none")

print(fig_its_by_stratum)
```

```r
# ============================================================
# PANEL ITS: Mean LOS (days) | CMF total vs hard vs soft
# Strata: sex (rows) x age group (cols)
# ============================================================

agg_year_sex_age_los_total <- trauma_base %>%
  dplyr::filter(
    is_cmf == 1,
    sexo_std %in% c("male","female"),
    !is.na(edad_cat), edad_cat %in% age_levels
  ) %>%
  dplyr::group_by(ANO_EGRESO, sexo_std, edad_cat) %>%
  dplyr::summarise(
    n_cmf    = dplyr::n(),
    n_los    = sum(!is.na(los)),
    mean_los = mean(los, na.rm = TRUE),
    sd_los   = sd(los, na.rm = TRUE),
    .groups  = "drop"
  ) %>%
  tidyr::complete(
    ANO_EGRESO = YEARS,
    fill = list(n_cmf = 0L, n_los = 0L, mean_los = NA_real_, sd_los = NA_real_)
  ) %>%
  dplyr::mutate(
    series   = "CMF total",
    sexo_std = factor(sexo_std, levels = c("male","female")),
    edad_cat = factor(edad_cat, levels = age_levels)
  )

agg_year_sex_age_los_bytype <- trauma_base %>%
  dplyr::filter(
    is_cmf == 1,
    cmf_group %in% c("hard","soft"),
    sexo_std %in% c("male","female"),
    !is.na(edad_cat), edad_cat %in% age_levels
  ) %>%
  dplyr::group_by(ANO_EGRESO, sexo_std, edad_cat, cmf_group) %>%
  dplyr::summarise(
    n_cmf    = dplyr::n(),
    n_los    = sum(!is.na(los)),
    mean_los = mean(los, na.rm = TRUE),
    sd_los   = sd(los, na.rm = TRUE),
    .groups  = "drop"
  ) %>%
  tidyr::complete(
    ANO_EGRESO = YEARS,
    fill = list(n_cmf = 0L, n_los = 0L, mean_los = NA_real_, sd_los = NA_real_)
  ) %>%
  dplyr::mutate(
    series = dplyr::case_when(
```

```r
      cmf_group == "hard" ~ "CMF hard",
      cmf_group == "soft" ~ "CMF soft",
      TRUE ~ NA_character_
    ),
    sexo_std = factor(sexo_std, levels = c("male","female")),
    edad_cat = factor(edad_cat, levels = age_levels)
  ) %>%
  dplyr::select(-cmf_group)


agg_year_sex_age_los_all <- dplyr::bind_rows(
  agg_year_sex_age_los_total,
  agg_year_sex_age_los_bytype
) %>%
  dplyr::mutate(series = factor(series, levels = c("CMF total","CMF hard","CMF soft")))


safe_fit_its_meanlos <- function(df) {
  if (sum(!is.na(df$mean_los)) < 6) return(NULL)
  tryCatch(
    fit_its_lm(df, y = "mean_los", transform = "none"),
    error = function(e) NULL
  )
}

its_pred_meanlos_strata <- agg_year_sex_age_los_all %>%
  dplyr::group_by(sexo_std, edad_cat, series) %>%
  dplyr::group_modify(~{
    res <- safe_fit_its_meanlos(.x)
    if (is.null(res)) return(tibble::tibble())
    res$pred
  }) %>%
  dplyr::ungroup()


fig_its_meanlos_by_stratum <- ggplot(its_pred_meanlos_strata, aes(x = ANO_EGRESO)) +
  geom_ribbon(aes(ymin = y_lo, ymax = y_hi, fill = series), alpha = 0.12, color = NA) +
  geom_line(aes(y = y_fit, color = series), linewidth = 1.0) +
  geom_point(aes(y = y_obs, color = series), size = 1.1, alpha = 0.6, na.rm = TRUE) +
  geom_vline(xintercept = c(2019, 2022), linetype = "dotted") +
  facet_grid(sexo_std ~ edad_cat, scales = "free_y") +
  labs(
    x = "Year",
    y = "Mean LOS (days)",
    color = "Series",
    fill  = "Series"
  ) +
  theme_minimal(base_size = 11) +
  theme(legend.position = "bottom") +
  guides(fill = "none")

print(fig_its_meanlos_by_stratum)
```

```r
# ============================================================
# PANEL STACKED AREA: Diagnostic mix within CMF HARD
# Strata: sex (rows) x age group (cols)
# ============================================================

cmf_hard_sex_age_year_diag <- cmf_base %>%
  dplyr::filter(
    cmf_group == "hard",
    sexo_std %in% c("male","female"),
    !is.na(edad_cat), edad_cat %in% age_levels,
    !is.na(DIAG1), DIAG1 != "",
    DIAG1 %in% CMF_HARD_SET
  ) %>%
  dplyr::count(ANO_EGRESO, sexo_std, edad_cat, DIAG1, name = "n") %>%
  tidyr::complete(
    ANO_EGRESO = YEARS,
    sexo_std = factor(c("male","female"), levels = c("male","female")),
    edad_cat = factor(age_levels, levels = age_levels),
    DIAG1 = factor(CMF_HARD_SET, levels = CMF_HARD_SET),
    fill = list(n = 0L)
  ) %>%
  dplyr::group_by(ANO_EGRESO, sexo_std, edad_cat) %>%
  dplyr::mutate(
    denom = sum(n),
    prop_within_hard = dplyr::if_else(denom > 0, n / denom, 0)
  ) %>%
  dplyr::ungroup() %>%
  dplyr::mutate(
    DIAG1 = factor(DIAG1, levels = CMF_HARD_SET),
    DIAG1_label = dplyr::recode(as.character(DIAG1), !!!hard_diag_labels),
    DIAG1_label = factor(DIAG1_label, levels = unname(hard_diag_labels))
  )

fig_hard_mix_panel <- ggplot(
  cmf_hard_sex_age_year_diag,
  aes(x = ANO_EGRESO, y = prop_within_hard, fill = DIAG1_label)
) +
  geom_area(alpha = 0.9, color = "white", linewidth = 0.15) +
  scale_fill_brewer(palette = "Accent") +
  geom_vline(xintercept = c(2019, 2022), linetype = "dotted") +
  scale_y_continuous(labels = scales::percent_format(accuracy = 1)) +
  facet_grid(sexo_std ~ edad_cat) +
  labs(
    x = "Year",
    y = "Composition within CMFt bone fractures (%)",
    fill = "Fractures of the skull and facial bones (ICD-10)"
  ) +
  theme_minimal(base_size = 11) +
  theme(legend.position = "bottom")

print(fig_hard_mix_panel)

# ============================================================
```

```r
# PANEL STACKED AREA: Etiology mix (6 groups) among KNOWN causes
# Strata: sex (rows) x age group (cols)
# ============================================================

etiol_missing_level <- "No especificada/otras"

etiol_counts_sex_age_year <- cmf_base %>%
  dplyr::filter(
    sexo_std %in% c("male","female"),
    !is.na(edad_cat), edad_cat %in% age_levels
  ) %>%
  dplyr::mutate(
    ext6 = as.character(ext_cause_6grp),
    ext6 = dplyr::if_else(is.na(ext6) | ext6 == "", etiol_missing_level, ext6),
    ext6 = dplyr::if_else(!(ext6 %in% c(etiol_6_levels, etiol_missing_level)), etiol_missing_level, ext
  ) %>%
  dplyr::count(ANO_EGRESO, sexo_std, edad_cat, ext6, name = "n") %>%
  tidyr::complete(
    ANO_EGRESO = YEARS,
    sexo_std = factor(c("male","female"), levels = c("male","female")),
    edad_cat = factor(age_levels, levels = age_levels),
    ext6 = factor(c(etiol_6_levels, etiol_missing_level), levels = c(etiol_6_levels, etiol_missing_leve
    fill = list(n = 0L)
  )


etiol_mix_sex_age_year_main <- etiol_counts_sex_age_year %>%
  dplyr::filter(ext6 %in% etiol_6_levels) %>%
  dplyr::group_by(ANO_EGRESO, sexo_std, edad_cat) %>%
  dplyr::mutate(
    denom_known = sum(n),
    pct = dplyr::if_else(denom_known > 0, 100 * n / denom_known, 0)
  ) %>%
  dplyr::ungroup() %>%
  dplyr::mutate(ext6 = factor(ext6, levels = etiol_6_levels))

fig_etiol_mix_panel <- ggplot(
  etiol_mix_sex_age_year_main,
  aes(x = ANO_EGRESO, y = pct, fill = ext6)
) +
  geom_area(alpha = 0.9, color = "white", linewidth = 0.15) +
  scale_fill_brewer(palette = "Set2") +
  geom_vline(xintercept = c(2019, 2022), linetype = "dotted") +
  scale_y_continuous(labels = scales::percent_format(scale = 1)) +
  facet_grid(sexo_std ~ edad_cat) +
  labs(
    x = "Year",
    y = "Proportion of CMF admissions (%)",
    title = "Etiological composition of CMF trauma by age group and sex",
    subtitle = "Percentages computed among admissions with known external cause (6-group classification
    fill = "Etiology (6 groups)"
  ) +
  theme_minimal(base_size = 11) +
```

```r
  theme(legend.position = "bottom")

print(fig_etiol_mix_panel)
```
