# Supplementary material for: Epidemiology of Craniomaxillofacial Trauma in Chile: A Systematic Review and 24-Year Nationwide Interrupted Time-Series Analysis
Source: Craniomaxillofac Trauma Reconstr. 2026 Jul 3;19(3):32. doi: 10.3390/cmtr19030032 (PMC13398010; doi:10.3390/cmtr19030032)
Supplement: Supplementary file 1 [file cmtr-19-00032-s001.zip › Supplementary-File S3.pdf]

Supplementary File 3

Supplementary figures and tables

Supplementary Table S1.Interrupted time series (negative binomial) model for national CMFt rates within hospitalized trauma discharges.

Term (interpretation)	beta (log-rate)	Robust SE (HC1)	IRR	95% CI (IRR)
Baseline rate (Year 2001; pre-COVID and pre-2022)	-2.914378	0.008975	0.054238	0.053292 to 0.055200
Annual secular trend (baseline slope; 2001–2019 counterfactual)	-0.008595	0.000793	0.991442	0.989902 to 0.992985
COVID period level change (2020–2021 vs counterfactual)	-0.127485	0.062308	0.880306	0.779105 to 0.994653
Post-acute level change (from 2022 onward vs counterfactual)	-0.162928	0.011653	0.849652	0.830466 to 0.869281
Post-2022 slope change (additional annual trend from 2022 onward)	0.025467	0.002944	1.025794	1.019893 to 1.031730

Supplementary Table S2. Interrupted time series (negative binomial) model for national CMFt rates within hospitalized trauma discharges (bone fractures).

Term (interpretation)	beta (log-rate)	Robust SE (HC1)	IRR	95% CI (IRR)
Baseline rate (Year 2001; pre-COVID and pre-2022)	-3.175529	0.012555	0.041772	0.040757 to 0.042813

Term (interpretation)	beta (log-rate)	Robust SE (HC1)	IRR	95% CI (IRR)
Annual secular trend (baseline slope; 2001–2019 counterfactual)	-0.004577	0.001165	0.995433	0.993164 to 0.997708
COVID period level change (2020–2021 vs counterfactual)	-0.137622	0.056208	0.871428	0.780524 to 0.972919
Post-acute level change (from 2022 onward vs counterfactual)	-0.143655	0.016756	0.866187	0.838202 to 0.895106
Post-2022 slope change (additional annual trend from 2022 onward)	0.017149	0.004182	1.017297	1.008993 to 1.025669

Supplementary Table S3. Interrupted time series (negative binomial) model for national CMFt soft-tissue injuries rates within hospitalized trauma discharges.

Term (interpretation)	beta (log-rate)	Robust SE (HC1)	IRR	95% CI (IRR)
Baseline rate (Year 2001; pre-COVID and pre-2022)	-4.374970	0.016853	0.012589	0.012180 to 0.013011
Annual secular trend (baseline slope; 2001–2019 counterfactual)	-0.024858	0.001808	0.975448	0.971998 to 0.978911
COVID period level change (2020–2021 vs counterfactual)	-0.090573	0.098759	0.913408	0.752661 to 1.108487
Post-acute level change (from 2022 onward vs counterfactual)	-0.286822	0.026356	0.750646	0.712853 to 0.790442
Post-2022 slope change (additional annual trend from 2022 onward)	0.066318	0.004375	1.068566	1.059442 to 1.077769

Supplementary Table S4. Segmented linear regression (interrupted time series) with injury-type interactions for mean length of hospital stay (LOS, days) after CMFT, 2001–2024.

Term (interpretation)	Estimate (days)	Robust SE (HC1)	t value	Pr(>
Baseline mean LOS for soft CMF (Year 2001; pre-COVID and pre-2022)	2.830668	0.169367	16.7132	< 2.2e-16
Baseline difference: hard vs soft CMF (Year 2001)	0.941546	0.192210	4.8985	1.822e-05
Annual secular trend for soft CMF (baseline slope; per year)	-0.049039	0.014062	-3.4873	0.001249
COVID period level change for soft CMF (2020–2021 vs counterfactual)	0.128119	0.157903	0.8114	0.422201
Post-acute level change for soft CMF (from 2022 onward vs counterfactual)	0.483564	0.245841	1.9670	0.056519
Post-2022 slope change for soft CMF (additional annual trend from 2022 onward)	0.110693	0.118570	0.9336	0.356421
Difference in annual secular trend: hard vs soft (baseline slope difference; per year)	0.057489	0.016158	3.5578	0.001023
Difference in COVID level change: hard vs soft (2020–2021)	0.176176	0.182636	0.9646	0.340830

Term (interpretation)	Estimate (days)	Robust SE (HC1)	t value	Pr(>
Difference in post-acute level change: hard vs soft (from 2022 onward)	0.018757	0.308522	0.0608	0.951841
Difference in post-2022 slope change: hard vs soft (additional annual trend from 2022 onward)	-0.261565	0.159248	-1.6425	0.108737

Supplementary Table S5. Interrupted time series (logit-linear segmented regression) for the proportion of bone fractures admissions with prolonged hospitalization (LOS ≥ 5 days), 2001–2024.

Term (interpretation)	OR	95% CI (OR)
Baseline odds of LOS 5 days (hard CMF; Year 2001)	0.339505	0.314977 to 0.365955
Annual secular trend (per year)	0.991884	0.985587 to 0.998221
COVID period level change (2020–2021)	1.152849	1.081820 to 1.228539
Post-acute level change (from 2022 onward)	1.216424	1.126441 to 1.313628
Post-2022 slope change (per year)	0.967622	0.943737 to 0.992111

Supplementary Table S6. Mean LOS according to the type of bone fracture, 2001–2024.

Diagnosis	Label	Mean annual LOS (days)	SD across years (days)
S020	Fracture of vault of skull	4.83	0.83
S022	Nasal bones fracture	2.08	0.17
S023	Orbital floor fracture	4.58	0.70
S024	Malar & maxillary fracture	4.81	0.22

Diagnosis	Label	Mean annual LOS (days)	SD across years (days)
S026	Mandibular fracture	5.31	0.59
S027	Multiple skull and facial bones fractures	8.35	0.93
S028	Other specified skull and facial bones fractures	5.44	0.96
S029	Unspecified facial fracture	5.45	0.80

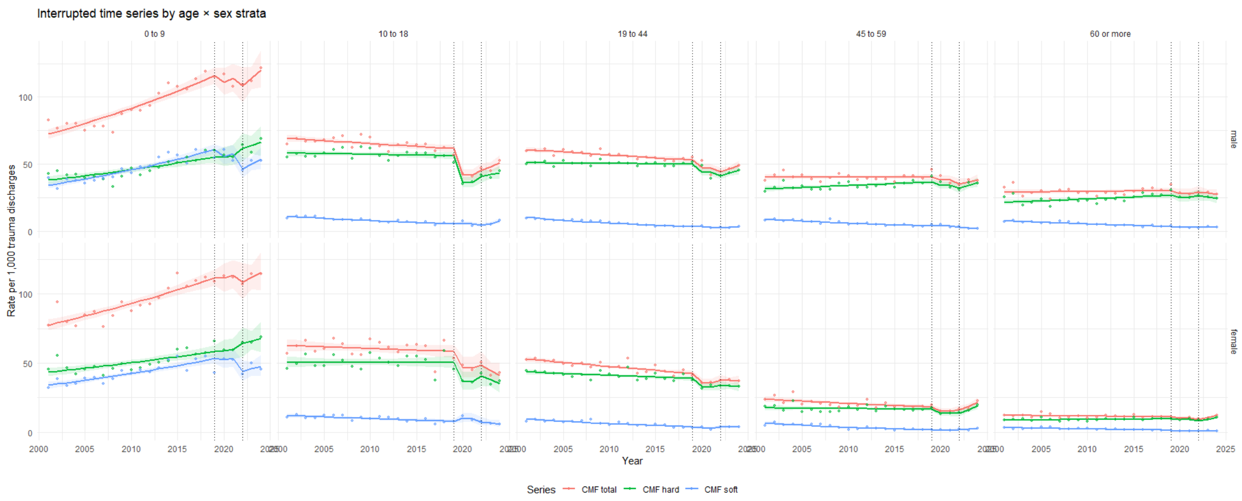

Supplementary Figure S1. Trends in rates according to age groups and sex (ITS)

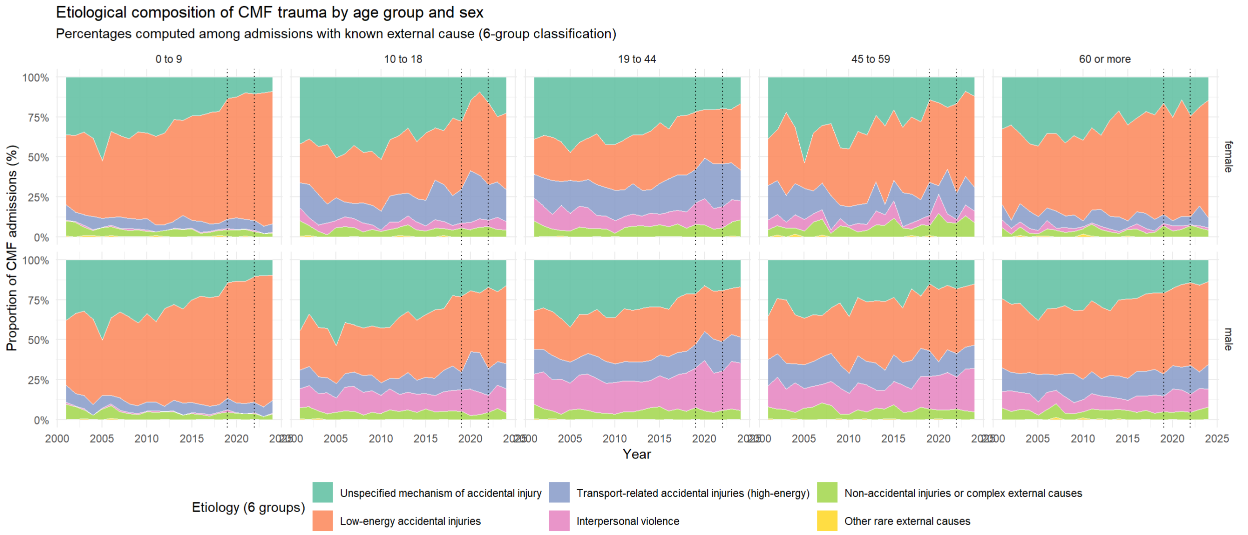

Supplementary Figure S2. Etiology composition within CMFt (bone fractures) according to age and sex

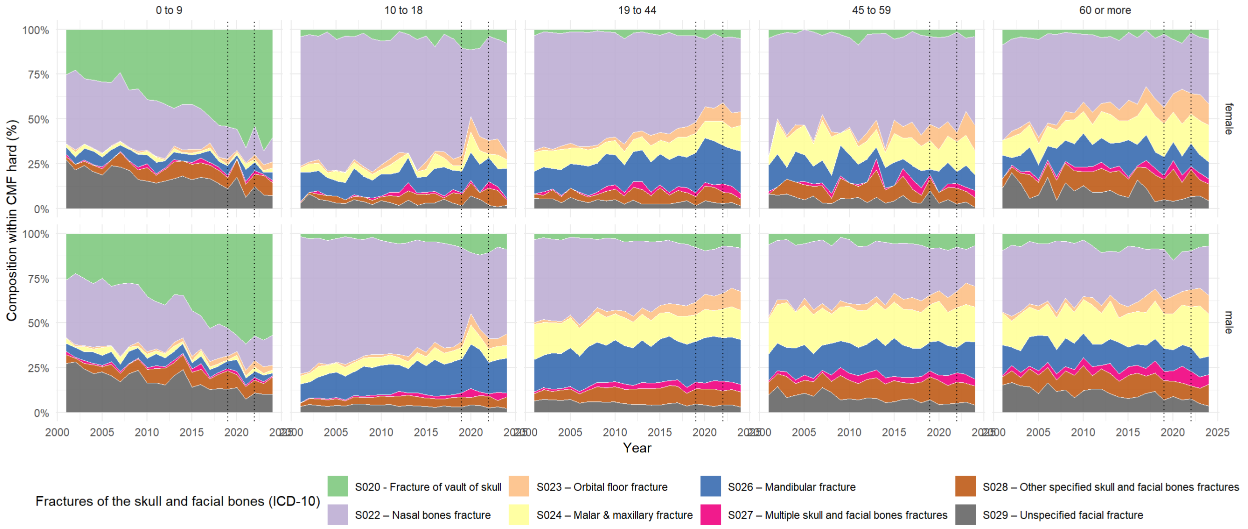

Supplementary Figure S3. Diagnostic composition within CMF fractures by age group and sex, 2001–2024.

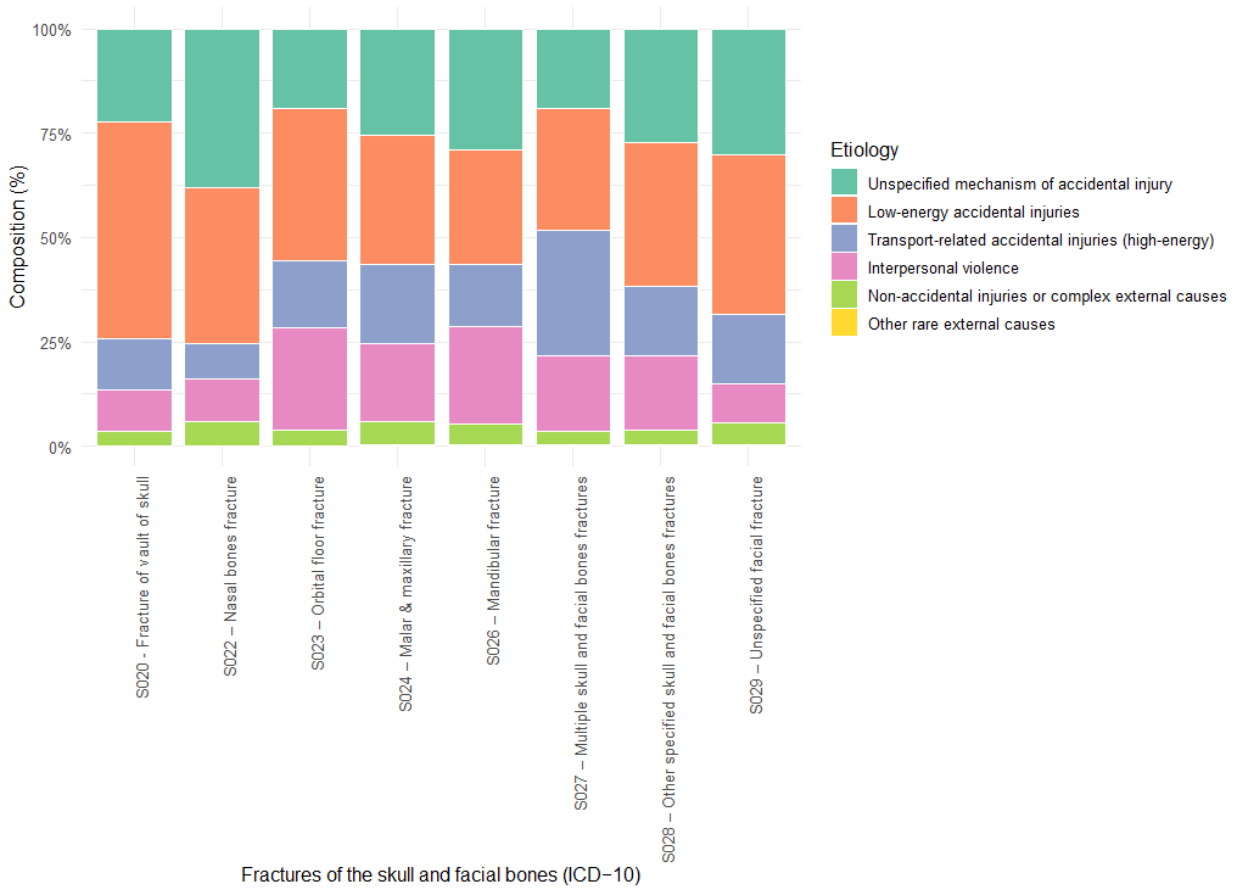

Supplementary Figure S4. Etiology composition within CMFt (bone fractures)

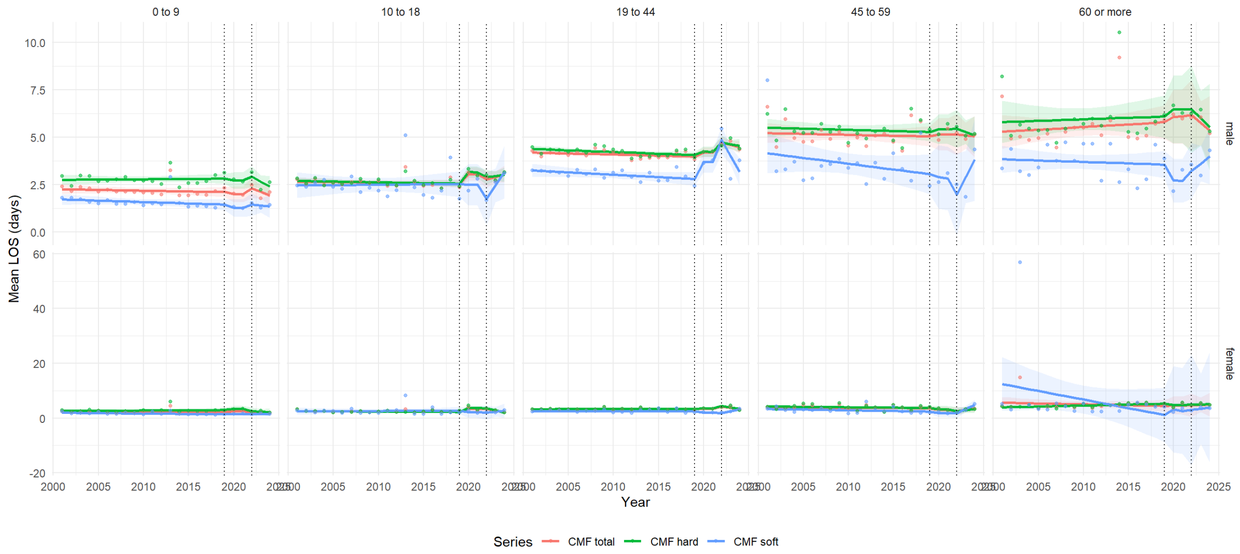

Supplementary Figure S5. Mean length of stay by age group and sex (CMFt admissions), 2001–2024.

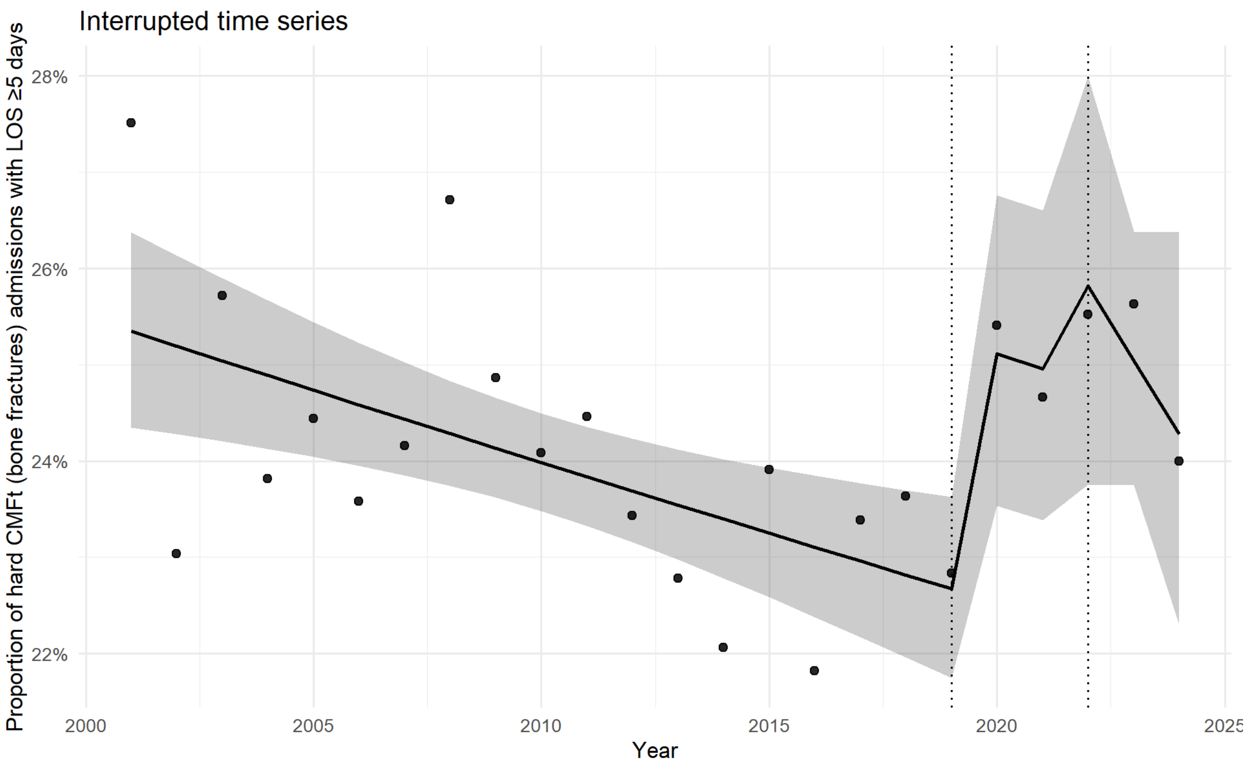

Supplementary Figure S6. Proportion of prolonged hospitalization (LOS ≥ 5 days) after bone fractures within CMFt (ITS)

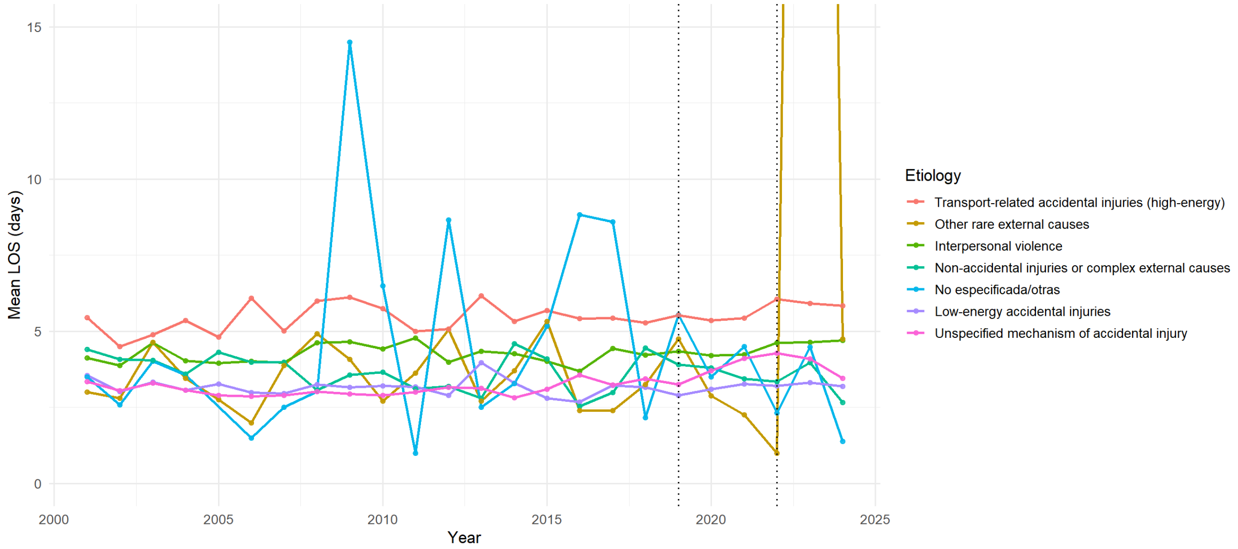

Supplementary Figure S7. Mean LOS of CMFt by etiology.
